# Comparative Analysis of Phenolic Compound Characterization and Their Biosynthesis Genes between Two Diverse Bread Wheat (*Triticum aestivum*) Varieties Differing for Chapatti (Unleavened Flat Bread) Quality

**DOI:** 10.3389/fpls.2016.01870

**Published:** 2016-12-15

**Authors:** Monica Sharma, Rajat Sandhir, Anuradha Singh, Pankaj Kumar, Ankita Mishra, Sanjay Jachak, Sukhvinder P. Singh, Jagdeep Singh, Joy Roy

**Affiliations:** ^1^National Agri-Food Biotechnology InstituteMohali, India; ^2^Department of Biochemistry, Panjab UniversityChandigarh, India; ^3^Department of Natural Products, National Institute of Pharmaceutical Education and ResearchMohali, India

**Keywords:** *Triticum aestivum*, phenolic compounds, UPLC-QTOF-MS and -MS/MS, Affymetrix wheat microarrays, qRT-PCR, phenylpropanoid pathway, Chapatti

## Abstract

Phenolic compounds (PCs) affect the bread quality and can also affect the other types of end-use food products such as chapatti (unleavened flat bread), now globally recognized wheat-based food product. The detailed analysis of PCs and their biosynthesis genes in diverse bread wheat (*Triticum aestivum*) varieties differing for chapatti quality have not been studied. In this study, the identification and quantification of PCs using UPLC-QTOF-MS and/or MS/MS and functional genomics techniques such as microarrays and qRT-PCR of their biosynthesis genes have been studied in a good chapatti variety, “C 306” and a poor chapatti variety, “Sonalika.” About 80% (69/87) of plant phenolic compounds were tentatively identified in these varieties. Nine PCs (hinokinin, coutaric acid, fertaric acid, p-coumaroylqunic acid, kaempferide, isorhamnetin, epigallocatechin gallate, methyl isoorientin-2′-O-rhamnoside, and cyanidin-3-rutinoside) were identified only in the good chapatti variety and four PCs (tricin, apigenindin, quercetin-3-O-glucuronide, and myricetin-3-glucoside) in the poor chapatti variety. Therefore, about 20% of the identified PCs are unique to each other and may be “variety or genotype” specific PCs. Fourteen PCs used for quantification showed high variation between the varieties. The microarray data of 44 phenolic compound biosynthesis genes and 17 of them on qRT-PCR showed variation in expression level during seed development and majority of them showed low expression in the good chapatti variety. The expression pattern in the good chapatti variety was largely in agreement with that of phenolic compounds. The level of variation of 12 genes was high between the good and poor chapatti quality varieties and has potential in development of markers. The information generated in this study can be extended onto a larger germplasm set for development of molecular markers using QTL and/or association mapping approaches for their application in wheat breeding.

## Introduction

Bread wheat (*Triticum aestivum* L.) is an important cereal crop whose flour is processed into several end-use food products such as bread, chapatti (unleavened flat bread), pasta, etc (Rao et al., 1986; Shewry, 2009). Phenolic compounds (PCs) in grains are important biomolecules as they affect bread-making quality (Sivam et al., 2011); contribute to quality parameters such as color, aroma, and taste (McCallum and Walker, 1989); and they have health benefits as they improve human health against diabetes, cardiovascular diseases, and associated diseases because of their high antioxidant properties (de Munter et al., 2007; Zilic et al., 2012a,b; Nicoletti et al., 2013). PCs affect the bread quality by interacting with the other biomolecules such as storage proteins and polysaccharides. It is presumed that they can also affect the other types of end-use food products such as chapatti (unleavened flat bread), which is now globally recognized wheat-based food product. The detailed analysis of PCs and their biosynthesis genes in diverse bread wheat (*T. aestivum*) varieties differing for chapatti quality have not been studied. In this study, the integrated approaches of analytical tools such as UPLC-QTOF-MS and -MS/MS and functional genomics techniques such as microarrays and qRT-PCR were used for the identification and quantification of PCs and quantitative expression analysis of their biosynthesis genes in two diverse bread wheat varieties differing for chapatti quality. The information generated in this study can be extended onto a larger wheat germplasm set for development of molecular markers using QTL and/or association mapping approaches for their application in wheat breeding.

PCs can be classified into different types depending on their structures and the detailed account on PCs and their measurement and application in food and health are described elsewhere (Cheynier, 2012). Briefly, liquid chromatography systems coupled with tandem time-of-flight mass spectrometer (LC-QTOF-MS or LC-QTOF-MS/MS) provide the faster characterization with better resolution and mass accuracy as compared to HPLC system (Segura-Carretero et al., 2008). HPLC coupled with UV detector has extensively been used for PC analysis (Jiang et al., 2013). However, some PCs, which did not have an absorption spectrum in UV-Visible light, cannot be identified such as quinic acid; cannot be separated such as galloylated catechins; and some PCs are difficult to quantify due to their very low concentration (Jiang et al., 2013). In comparison to HPLC, UPLC coupled with tandem mass spectrometer (UPLC-QTOF-MS or MS/MS) provides faster analysis in low concentration with better resolution, short run time, and high quality ion spectra. It can detect additional compounds and fewer isobars as compared to HPLC (Evans et al., 2009).

**Figure 1:**
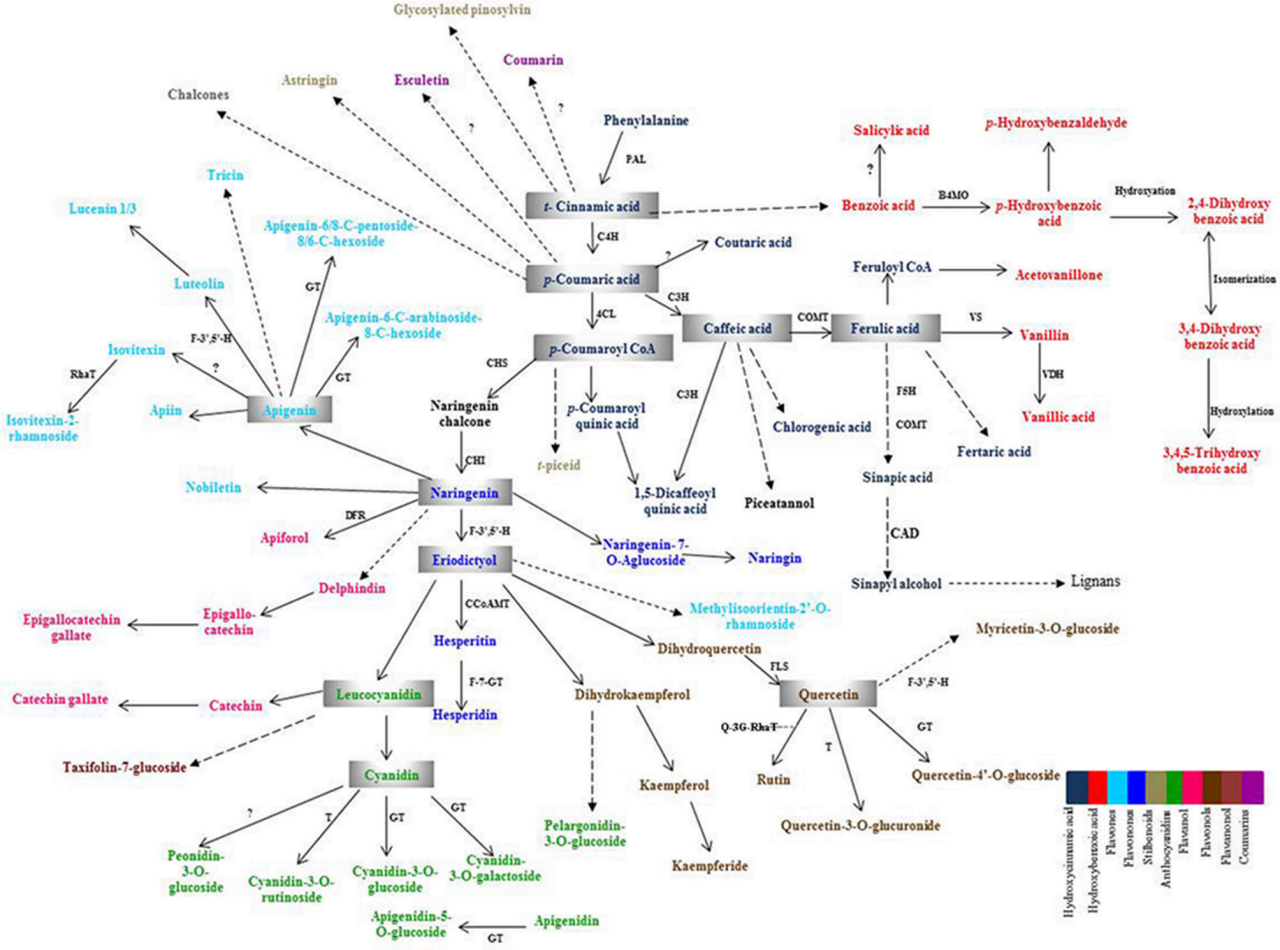
**Schematic overview of phenolic compounds biosynthesis pathway**. PAL, Phenylalanine ammonia lyase; C4H, Cinnamate-4-hydoxylase; 4CL, 4-Coumarate CoA ligase; C3H, *p*-Coumaroyl shikimate 3-hydroxylase; COMT, Caffeic acid-O-methyltransferase; F5H 1, Ferulate-5-hydroxylase 1; CCoAMT, Caffeoyl CoA-O-methyl transferase; B4MO, Benzoate-4-monooxygenase; VS, Vanillin synthase; VDH, Vanillin dehydrogenase; CAD, Cinnamyl alcohol dehydrogenase; DFR, Dihydroflavonol 4- reductase; GT, Glucosyl transferase; RhaT, Rhamnosyl Transferase; F-7-GT, Flavanone 7-O-beta-glucosyltransferase; F-3′,5′-H, Flavonoid 3′,5′-hydroxylase; CHS, Chalcone synthase; CHI, Chalcone isomerase; FLS, Flavonol synthase; T, Transferase, Q3G-RhaT, Quercetin-3-O-glucoside L-rhamnosyltransferase.

Plant PCs are biosynthesized via shikimic acid pathway, phenylpropanoid pathway, and flavonoid pathway (Herrmann and Weaver, 1999; Jiang et al., 2013). The precursor biomolecule, phenylalanine, an aromatic amino acid, is deaminated in presence of phenylalanine ammonia lyase (PAL), which is a key enzyme of this pathway to produce *t*-cinnamic acid. *t*-cinnamic acid is further acted upon by the enzyme, cinnamate-4-hydroxylase (C4H), to produce *p*-coumaric acid and then give rise to all other phenolic compounds and their derivatives such as phenolic acids, flavonoids, coumarins, lignans, etc. The schematic diagram showing biosynthesis of major PCs and their enzymes is provided in Figure 1. The temporal and spatial distribution of expression of plant PCs biosynthesis genes is limited in wheat in comparison to model plant species such as *Arabidopsis* and tobacco. Expression analysis of plant PCs biosynthesis pathway genes during plant growth and seed development will provide the information on their tissue specific expression as well as their spatial and temporal expression level.

In this study, two diverse bread wheat (*T. aestivum*) varieties, “C 306,” a traditionally known good chapatti quality variety and “Sonalika,” a poor chapatti quality variety (Bhatnagar et al., 2002) were used for the identification and quantification of plant PCs and expression analysis of their biosynthesis genes between them. The comparative data analysis showed that the poor chapatti quality wheat variety, “Sonalika” has higher concentration of majority of PCs in comparison to that of the good chapatti quality variety and also identified inter-variety specific PCs. The information generated in this study will be used in a large wheat germplasm set for developing DNA-based markers for marker-assisted breeding of specific phenolic compounds as well as their manipulation through functional genomics tools such as RNAi and genome editing. This study enlightened the scope for improvement of PCs-based processing and nutrition quality in wheat varieties through molecular breeding approaches.

## Materials and methods

### Plant materials

The mature seeds (14% moisture level) of two diverse bread wheat (*T. aestivum*) varieties, “C 306” (good) and “Sonalika” (poor) differing for chapatti (unleavened flat bread) quality were used for the characterization and quantification of phenolic compounds, while their developing seeds were used for quantitative expression analysis of their biosynthesis pathway genes. The details of the two varieties including pedigree and other information were described elsewhere (Singh et al., 2014). The seeds of above varieties were completely dried at 60°C for at least 6 h before extraction of phenolic compounds.

### Extraction of phenolic compounds

The phenolic compounds were analyzed in three different types of extracts-free (soluble), bound-alkali, and bound-acidic extracts of wheat grains in three replicates. The free (soluble) and bound phenolic compounds were extracted, following the protocol of Adom et al. (2003), with modification. Adom et al. (2003) used both diethyl ether and ethyl acetate for extraction of bound phenolic compounds whereas we used only ethyl acetate for extracting bound phenolics. The final extraction of PCs was done with ethyl acetate and PCs were reconstituted in 80% methanol and dried in a rotary evaporator. The extracted PCs were filtered through a 0.22 μm nylon syringe filter, and stored at −20°C.

### Preparation of samples for analysis

Each sample extract was dissolved in 2 ml of 80% (v/v) methanol, filtered through a 0.22 μm nylon syringe filter, and stored at −20°C till injecting into the UPLC system.

### Preparation of standard solutions for analysis

Stock solution for each standard was prepared to final concentration of 1.0 mg/ml, filtered through 0.22 μm syringe filter, and stored at −20°C till use. Each standard was sonicated before injecting into the UPLC for analysis. The solution with the highest concentration was prepared by taking 3 μl of each standard and making the final concentration of 3 μg/ml. Different dilutions ranging from 0.005 to 3 μg/ml of each standard solution were prepared from the stock standard mixture for the preparation of linear standard curve.

### Chromatographic conditions on UPLC-QTOF

Phenolic compounds were separated on an ACQUITY™ UPLC system equipped with a binary solvent manager and sample manager (Waters, Milford, MA, USA). The method was developed on an ACQUITY UPLC® BEH C18 column (2.1 × 50 mm, 1.7 μm) equipped with an ACQUITY UPLC BEH guard column containing 0.2 μm metal frit coupled with a TripleTOF® 5600 mass spectrometer (ABSciex, Massachusetts, USA). The spectrometer is a quadrupole-time-of-flight (Q-TOF) which is used for high-resolution quantitative and qualitative analysis of compounds.

The mobile phase consisted of 0.1% formic acid in ACN (solvent A) and 0.1% formic acid in Milli-Q water (solvent B). The gradient for separation was performed as 0–1 min solvent A, 99%; 1–2 min solvent A, 99%; 2–16 min solvent A, 1%; and 16–18 min column equilibration with solvent A, 99% and 18–20 min solvent A, 99%. The flow rate of 300 μl/min was used for separation. MS/MS analysis was done for the fragmentation and identification of the molecules in the sample. Data processing was performed using Analyst TF software (version 1.5.1) and quantification was carried out using Multiquant software (version 2.0.2) and matching was carried out using Peakview software (version 1.2.0.3). The dwell time and scan cycles was automatically set by the software.

### Method validation for quantification of PCs

The UPLC method used in this study for the identification and quantification of PCs was validated by carrying out linearity, limit of detection (LOD), limit of quantification (LOQ), and precision following the International Conference on Harmonization [(ICH Q2(R1)] guidelines (ICH, 2005).

#### Linearity, LOD, LOQ, and precision

Linearity was determined by preparing calibration curves from five different concentrations for each standard separately. The calibration curve was used to calculate the regression coefficient, slope, and intercept and quantify the respective compounds in the two wheat varieties. Following the guidelines, LOD and LOQ of each standard were determined based on the signal to noise (S/N) ratios of 3 and 10, respectively. The intra-day and inter-day precisions were used to evaluate reproducibility and repeatability of the method of quantification. For intra-day precision, each standard was analyzed at three different concentrations (50, 500, and 1000 ng/ml) in triplicate within 1 day. For inter-day precision, each standard was analyzed at the same three different concentrations in triplicate for 3 consecutive days and the precision data were expressed as relative standard deviation (RSD %).

### Expression analysis of phenolic compound biosynthesis pathway genes using microarray data

The expression data of ninety-one probe sets of forty-one genes, which were relevant to phenolic compound biosynthesis pathway, were extracted from our publicly available gene expression data of Affymetrix® Wheat Genome Arrays on the above two bread wheat varieties, “C 306” and “Sonalika” along with the other two bread wheat varieties, “LOK1”(good chapatti quality) and “WH 291” (poor chapatti quality; Singh et al., 2014). The expression data of the four varieties were analyzed for expression potential (heatmap) in three seed developmental stages (7, 14, and 28 days after anthesis) in the software, GENEVESTIGATOR®. The expression data of the two varieties, “C 306” and “Sonalika,” which were used for phenolic compound characterization, were extracted from the published microarray data for differential expression analysis between the good and poor chapatti quality varieties.

### Quantitative real time gene expression analysis using qRT-PCR

The detail protocols of RNA extraction, cDNA synthesis, and quantitative gene expression analysis are described elsewhere (Singh et al., 2014). The primer pairs for quantitative gene expression analysis were designed using Primer Express Software Tool ver. 3.0 (Applied Biosystems, Forster City, CA, USA). The nucleotide sequences of the pathway genes for primer designing were extracted from the GeneChip® Wheat Genome Array (Affymetrix, Santa Clara, USA). ADP- ribosylation factor (ARF) was used as a house-keeping gene for normalization of gene expression. The gene expression analysis was calculated using the method described in Livak and Schmittgen (2001).

### Data analysis

Analysis of the molecular ions (MS) at individual peak and the fragments of their main ion of PCs generated by UPLC-QTOF-MS/MS were done by matching the RT, MS, MS/MS fragmentation patterns of their standards. The chemical formulae and the molecular masses of 87 phenolic compounds (Supplementary Table 1) reported in plants were submitted in the Peakview software (version 1.2.0.3). PCs were validated and quantified using their standards. Quantification was done in triplicates for the three extracts of each variety using Multiquant software (version 2.0.2).

## Results

### Tentative identification of phenolic compounds on UPLC-QTOF-MS in good and poor chapatti (unleavened flat bread) quality bread wheat varieties

**Figure 2:**
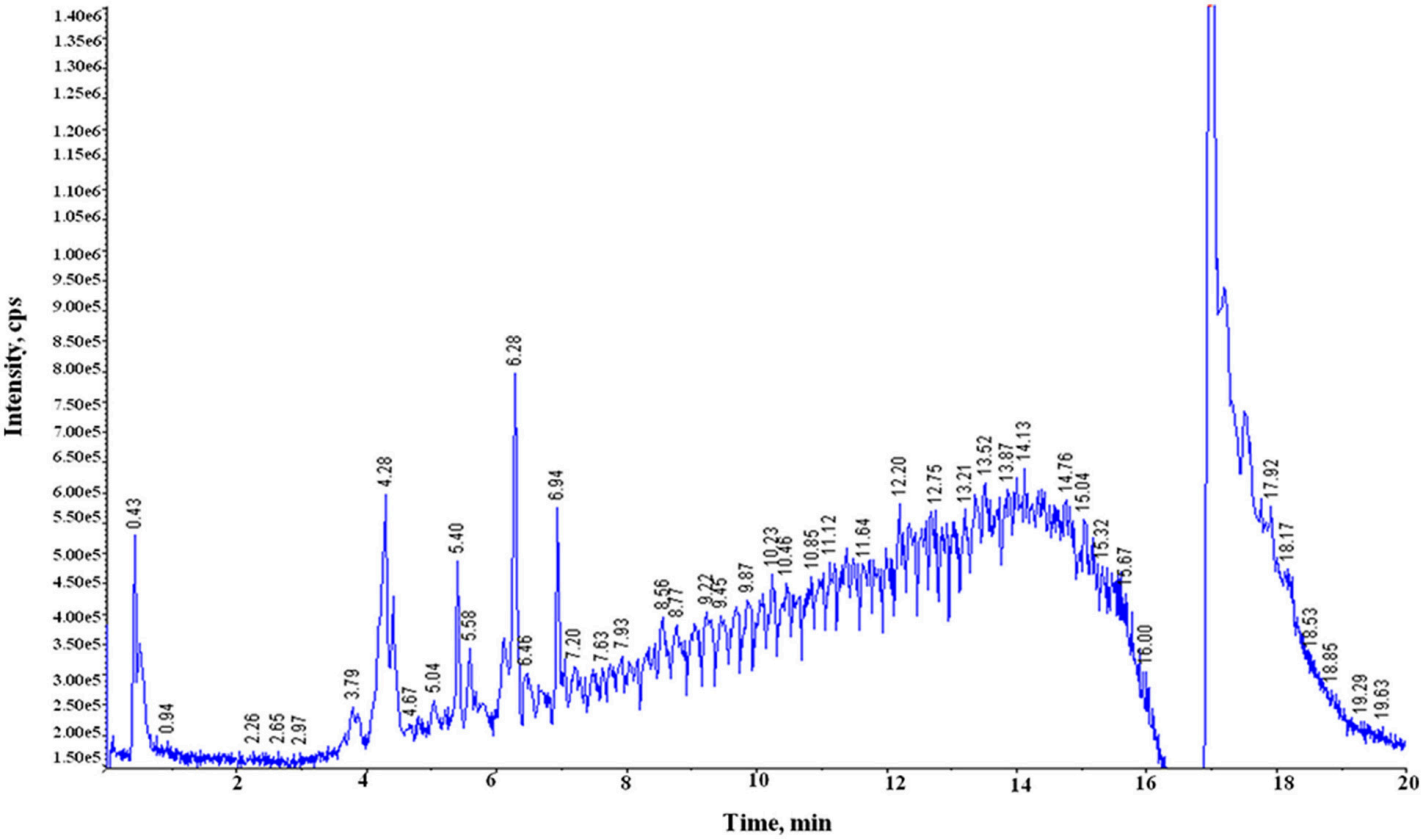
**Total ion chromatograms (TIC) generated on UPLC-QTOF-MS showing peaks of phenolic compounds in the acidic extract of the poor chapatti (unleavened flat bread) bread wheat variety, “Sonalika.”** Y-axis and X-axis represent **s**ignal intensity (counts per second, cps) and retention time (RT), respectively.

Both free (soluble) and bound forms of PCs from six samples of the two diverse wheat varieties for chapatti quality were fractionated, and the bound PCs were further fractionated into alkali-labile and acid-labile components by successive alkaline and acid treatments. Sixty-nine of eighty-seven plant PCs (~79%) were tentatively identified in these extracts by comparing their mass determined on UPLC-QTOF-MS with that of the previously known mass data of the plant PCs (Table 1 and Supplementary Table 1). The UPLC chromatograms of the six extracts were provided in Figure 2 and Supplementary Figure 1. About 50% of plant PCs were detected in all six samples and the remaining PCs were detected in at least one sample. The 69 PCs were grouped into 23 phenolic acids and their derivatives including lignans, 38 flavonoids including anthocyanidins, 4 stilbenoids, 2 chalcones, and 2 coumarins (Table 1).

**Table 1:** **Tentative identification of phenolic compounds by matching their mass with that of plants PCs using Peakview software (version 1.2.0.3)**.

**S. no**.	**RT (min)**	**Molecular formula**	**Molecular mass**	**Experimental mass**	**Polarity**	**Phenolic compound (PC)**	**Class of PC**	**Extract type**		
								**Free**	**Alkaline**	**Acidic**
1	0.02	C_20_H_18_O_6_	354.35	355.35	[M+H]^+^	Hinokinin	Lignan	ND	C	C
2	1.04	C_7_H_6_O_5_	170.12	169.12	[M−H]^−^	Gallic acid	HBA	S	S	C,S
3	3.08	C_7_H_6_O_3_	138.12	137.12	[M−H]^−^	*p*-Hydroxy benzoic acid	HBA	C,S	C,S	C,S
4	3.09	C_15_H_12_O_6_	288.25	287.25	[M−H]^−^	Eriodictyol	Flavanone	C	C,S	C
5	3.78	C_7_H_6_O_4_	154.12	153.12	[M−H]^−^	2,4-Dihydroxybenzoic acid	HBA	C,S	C	C
6	3.79	C_7_H_6_O_4_	154.12	153.12	[M−H]^−^	3,4-Dihydroxy benzoic acid	HBA	C,S	C	C
7	4.05	C_7_H_6_O_2_	122.12	121.12	[M−H]^−^	Benzoic acid	Phenolic acid	C,S	C,S	C,S
8	4.38	C_13_H_12_O_8_	296.23	295.23	[M−H]^−^	Coutaric acid	HCA	ND	ND	C
9	4.4	C_9_H_6_O_4_	178.14	177.14	[M−H]^−^	Esculetin	Coumarin	C,S	C,S	C
10	4.41	C_9_H_8_O_4_	180.15	179.15	[M−H]^−^	Caffeic acid	HCA	C,S	C,S	C,S
11	4.54	C_14_H_14_O_9_	326.25	325.25	[M−H]^−^	Fertaric acid	HCA	ND	ND	C
12	4.82	C_16_H_18_O_8_	338.30	337.30	[M−H]^−^	*p*-coumaroylquinic acid	HCA	C	C	C
13	4.9	C_21_H_24_O_10_	436.40	435.40	[M−H]^−^	Phloridzin	Chalcone	C,S	C,S	C,S
14	5.03	C_10_H_10_O_4_	194.18	193.18	[M−H]^−^	Ferulic acid	HCA	C,S	C,S	C,S
15	5.03	C_8_H_8_O_4_	168.14	167.13	[M−H]^−^	Vanillic Acid	HBA	C,S	C,S	C
16	5.06	C_9_H_8_O_2_	148.15	147.15	[M−H]^−^	*t*-Cinnamic acid	Phenolic acid	C,S	C,S	C,S
17	5.11	C_20_H_22_O_8_	390.39	389.39	[M−H]^−^	*t*-piceid	Stilbenoid	ND	ND	C,S
18	5.11	C_9_H_8_O_3_	164.16	163.16	[M−H]^−^	*p*-Coumaric acid	HCA	C,S	S	C,S
19	5.18	C_16_H_18_O_9_	354.31	353.31	[M−H]^−^	Chlorogenic acid	HCA	C,S	C,S	C,S
20	5.2	C_9_H_6_O_2_	146.14	147.14	[M+H]^+^	Coumarin	Coumarin	C,S	C,S	C,S
21	5.41	C_21_H_20_O_11_	448.37	447.37	[M−H]^−^	Orientin/Isoorientin	Flavone	C,S	ND	C,S
22	5.41	C_27_H_30_O_16_	610.51	609.51	[M−H]^−^	Rutin	Flavonol	C,S	C,S	C
23	5.45	C_11_H_14_O_4_	210.22	209.22	[M−H]^−^	Sinapyl alcohol	HCA	C,S	C,S	C,S
24	5.56	C_8_H_8_O_3_	152.15	151.15	[M−H]^−^	Vanillin	HBA	C,S	C,S	C,S
25	5.58	C_11_H_12_O_5_	224.21	223.21	[M−H]^−^	Sinapic acid	HCA	C,S	C,S	C,S
26	5.77	C_15_H_12_O_5_	272.25	271.25	[M−H]^−^	Naringenin	Flavanone	C,S	C,S	C,S
27	5.77	C_25_H_24_O_12_	516.45	517.45	[M+H]^+^	1,5-Dicaffeoyl quinic acid	HCA	C,S	C,S	C,S
28	5.77	C_27_H_30_O_15_	594.52	595.52	[M+H]^+^	Apigenin-6/8-C-pentoside-8/6-C-hexoside	Flavone	S	C,S	C,S
29	5.84	C_28_H_34_O_15_	610.57	611.57	[M+H]^+^	Hesperidin	Flavanone	C,S	C,S	C,S
30	6.01	C_21_H_20_O_12_	464.37	465.37	[M+H]^+^	Quercetin-4′-O-glucoside	Flavonol	C,S	C,S	C
31	6.08	C_21_H_22_O_10_	434.39	435.39	[M+H]^+^	Naringenin-7-O-glucoside	Flavanone	C,S	C,S	C,S
32	6.09	C_7_H_6_O_3_	138.12	139.12	[M+H]^+^	Salicylic acid	HBA	C,S	C,S	C,S
33	6.15	C_15_H_14_O_5_	274.26	275.26	[M+H]^+^	Phloretin	Chalcone	C,S	C,S	C,S
34	6.23	C_21_H_22_O_8_	402.39	403.39	[M+H]^+^	Nobiletin	Flavone	C,S	C,S	C,S
35	6.24	C_15_H_11_O_6_	287.24	288.24	[M+H]^+^	Cyanidin	Anthocyanidin	C,S	ND	C,S
36	6.41	C_15_H_14_O_5_	274.26	275.26	[M+H]^+^	Apiforol	Flavanol	C,S	C,S	C,S
37	6.96	C_15_H_10_O_7_	302.23	303.23	[M+H]^+^	Quercetin	Flavonol	C,S	C,S	C,S
38	7.22	C_15_H_10_O_6_	286.23	287.23	[M+H]^+^	Luteolin	Flavone	C,S	ND	C,S
39	7.26	C_16_H_12_O_6_	300.26	299.26	[M−H]^−^	Kaempferide	Flavonol	ND	ND	C
40	7.58	C_9_H_10_O_3_	166.17	165.17	[M−H]^−^	Acetovanillone	HBA	C,S	C,S	C,S
41	7.77	C_15_H_10_O_6_	286.23	285.23	[M−H]^−^	Kaempferol	Flavonol	C,S	S	C,S
42	8.17	C_14_H_12_O_4_	244.25	245.25	[M+H]^+^	Piceatannol	Stilbenoid	C,S	C,S	C,S
43	8.2	C_16_H_12_O_7_	316.26	315.26	[M+H]^+^	Isorhamnetin	Flavonol	C	C	C
44	8.56	C_21_H_21_O_9_	417.38	416.38	[M−H]^−^	Apigenindin-5-O- glucoside	Anthocyanidin	C,S	C,S	C,S
45	8.63	C_20_H_22_O_9_	406.38	407.38	[M+H]^+^	Astringin	Stilbenoid	C,S	C,S	C,S
46	8.84	C_27_H_30_O_14_	578.52	579.52	[M+H]^+^	Isovitexin-2′-O-rhamnoside	Flavone	C,S	C,S	C,S
47	9.17	C_20_H_20_O_7_	372.37	373.37	[M+H]^+^	Sinensetin	Flavone	C,S	ND	ND
48	12.21	C_22_H_23_O_11_Cl	498.87	499.87	[M+H]^+^	Peonidin-3-glucoside	Anthocyanidin	C,S	S	C,S
49	12.21	C_21_H_21_O_10_Cl	468.84	467.84	[M−H]^−^	Pelargonidin-3-glucoside (callistephin)	Anthocyanidin	ND	S	C
50	12.23	C_15_H_14_O_6_	290.26	289.26	[M−H]^−^	Catechin	Flavanol	C,S	C,S	S
51	13.13	C_21_H_21_O_11_	449.38	448.38	[M−H]^−^	Cyanidin-3-galactoside	Anthocyanidin	C,S	C,S	C,S
52	13.13	C_21_H_22_O_8_	402.39	401.39	[M−H]^−^	Glycosylated pinosylvin	Stilbenoid	C,S	C,S	C,S
53	13.14	C_23_H_24_O_12_	492.43	491.43	[M−H]^−^	Glycosylated 3′,4′,5′-trihydroxy-3,7-dimethylflavone	Flavone	C,S	S	C,S
54	13.15	C17H14O7	330.29	331.29	[M+H]^+^	Tricin	Flavone	S	ND	ND
55	13.16	C_15_H_11_O_4_	255.24	256.24	[M+H]^+^	Apigenindin	Anthocyanidin	S	ND	ND
56	13.16	C_22_H_18_O_10_	442.37	443.37	[M+H]^+^	Epigallocatechin gallate	Flavanol	ND	ND	C
57	13.69	C_26_H_28_O_14_	564.49	563.49	[M−H]^−^	Apiin	Flavone	C,S	C,S	C,S
58	14.05	C_21_H_22_O_12_	466.39	465.39	[M−H]^−^	Taxifolin-7-glucoside	Flavanonol	C,S	C,S	C,S
59	14.14	C_21_H_18_O_13_	478.35	479.35	[M+H]^+^	Quercetin-3-O-glucuronide	Flavonol	S	S	ND
60	14.16	C_22_H_18_O_11_	458.37	459.37	[M+H]^+^	Catechin gallate	Flavanol	C	ND	ND
61	14.31	C_28_H_32_O_15_	608.54	607.54	[M−H]^−^	Methylisoorientin-2′-O-rhamnoside	Flavone	ND	ND	C
62	14.31	C_26_H_28_O_14_	564.49	563.49	[M−H]^−^	Apigenin-6-C-arabinoside-8-C-hexoside	Flavone	C,S	C,S	C,S
63	15.07	C_21_H_20_O_13_	480.37	481.37	[M+H]^+^	Myricetin-3-glucoside	Flavonol	ND	S	ND
64	15.33	C_22_H_26_O_8_	418.43	419.43	[M+H]^+^	Syringaresinol	Lignan	C	ND	ND
65	15.45	C_27_H_32_O_14_	580.34	581.34	[M+H]^+^	Naringin	Flavanone	C	C,S	ND
66	15.6	C_7_H_6_O_2_	122.12	123.12	[M+H]^+^	*p*-Hydroxybenzaldehyde	HBA	C,S	C,S	C,S
67	15.91	C_26_H_28_O_15_	580.49	581.49	[M+H]^+^	Luteolin-6/8-C-xyloside-8/6-C-glucoside	Flavone	S	ND	C
68	16.11	C_27_H_31_O_15_	595.52	594.52	[M−H]^−^	Cyanidin-3-rutinoside	Anthocyanidin	C	C	C
69	16.22	C_21_H_21_O_11_	449.38	448.38	[M−H]^−^	Cyanidin-3-glucoside (kuromanin)	Anthocyanidin	C,S	C,S	C,S

*RT, Retention time; ND, Not detected; C, “C 306” a good chapatti (unleavened flat bread) bread wheat variety; S, “Sonalika” a poor chapatti bread wheat variety*.

*The detail of plant PCs is provided in Supplementary Table 1*.

#### Phenolic acids and their derivatives

Twenty-three phenolic acids and their derivatives were identified in the six samples (Table 1) and they were further sub-grouped into two phenolic acids (benzoic acid and cinnamic acid), nine hydroxybenzoic acids (HBA), ten hydroxycinnamic acids (HCA), and two lignans (hinokinin and syringaresinol). In HBA sub-group, nine hydroxy derivatives of benzoic acid were identified in the samples. These were two mono-hydroxy benzoic acids (*2*-hydroxy benzoic acid or salicylic acid and *4*-hydroxy benzoic acid or *p*-hydroxy benzoic acid), two dihydroxy benzoic acids (*2,4*-dihhydroxy benzoic acid and *3,4*-dihhydroxy benzoic acid), one trihydroxy benzoic acid (*3,4,5*-trihhydroxy benzoic acid or gallic acid), one methoxy derivative (*4*-hydroxy-*3*-methoxy benzoic acid (or vanillic acid), two aldehyde derivatives (*4*-hydroxy-*3*-methoxy benzaldehyde or vanillin and *4*-hydroxy benzaldehyde or *p*-hydroxy benzaldehyde), and 1-(*4*-hydroxy-*3*-methoxyphenyl) ethanone or acetovanillone (structurally similar to vanillin).

In HCA sub-group, four simple phenolic compounds such as *p*-coumaric acid, caffeic acid, ferulic acid, and sinapic acid were identified in the samples. Three quinic acid derivatives of hydroxycinnamic acids (HCAs) such as chlorogenic acid (an ester of caffeic acid and quinic acid), *p*-coumaroylquinic acid (an ester of *p*-coumaric acid and quinic acid), and 1,5-dicaffeoylquinic acid (an ester of two units of caffeic acids and quinic acid) were identified in the samples. Two tartaric acid derivatives (ester derivatives) of HCAs such as fertaric acid (ferulic acid with tartaric acid) and coutaric acid (*p*-coumaric acid with tartaric acid) were identified in the samples. A monolignol such as sinapyl alcohol (an alcohol derivative of HCA) was identified in the samples. It is a precursor for biosynthesis of both lignan and lignin (Vanholme et al., 2010).

#### Flavonoids

In this study a total of 38 flavonoids were identified in the six samples (Table 1). The 38 flavonoids were classified into five sub-groups—8 flavonols, 12 flavones, 5 flavanones, 4 flavanols, 1 flavanonol (Taxifolin-7-glucoside), and 8 anthocyanidins. Eight flavonols (having 3-hydroxyflavone backbone) such as three aglycones (quercetin, kaempferide, and isorhamnetin), two O-glycosylated compounds (quercetin-3-*O*-rutinoside or rutin, myricetin-3-glucoside, and quercetin-4′-*O*-glucoside), quercetin-3-O-glucuronide (glucuronic acid derivative), and kaempferol were identified in the samples. Twelve flavones such as luteolin (one hydroxyl derivative), eight glycosylated flavones (orientin/isoorientin or luteolin-6-C-glucoside, apigenin-6/8-C-pentoside-8/6-hexoside, Isovitexin-2′-O-rhamnoside, apiin, methyl isoorientin-2′-O-rhamnoside isomer, apigenin-6-C-arabinoside-8-C-hexoside, luteolin-6/8-C-xyloside-8/6-C-glucoside or lucenin-1/3 isomer, and glycosylated 3′,4′,5′-trihydroxy-3,7-dimethyl flavone isomer), and three methylated flavones (nobiletin, sinensetin, and tricin) were identified in the samples. Most of flavones are glycosylated. Five flavanones such as 5,7-dihydroxy-2-(4-hydroxy phenyl) chroman-4-one, eriodictyol, naringenin, naringenin-7-*O*-glucoside, and hesperidin were identified in the samples. Four flavanols such as three flavan-3-ols (catechin, catechin gallate, and epigallocatechin gallate) and one flavan-4-ol (apiforol) were identified in the samples. In this study, eight anthocyanidins such as apigenindin, cyanidin, and six gylcosides (apigenindin-5-O-glucoside, peonidin-3-glucoside, pelargonidin-3-glucoside or callistephin, cyanidin-3-galactoside, cyanidin-3-rutinoside, and cyanidin-3-glucoside) were identified in the samples.

#### Chalcones, stilbenoids, coumarins

Chalcone is an aromatic ketone that forms central core of many biomolecules. In this study, two chalcone compounds—phloretin and phloridzin were identified in the six wheat samples. Stilbenoid has a C6-C2-C6 backbone structure. Four stilbenoid compounds—piceatannol, astringin, *t*-piceid, and glycosylated pinosylvin were identified in this study. Piceatannol is aglycone whereas astringin and *t*-piceid are glycosides of piceatannol and *t*-resveratrol, respectively. Coumarins belong to benzopyrone chemical class and have vanilla like fragrance. In this study two coumarins such as 6,7-dihydroxy-chromen-2-one or esculetin and 2H-chromen-2-one or coumarin were identified.

### Validation of phenolic compounds using their standards on UPLC-QTOF-MS/MS

**Figure 3:**
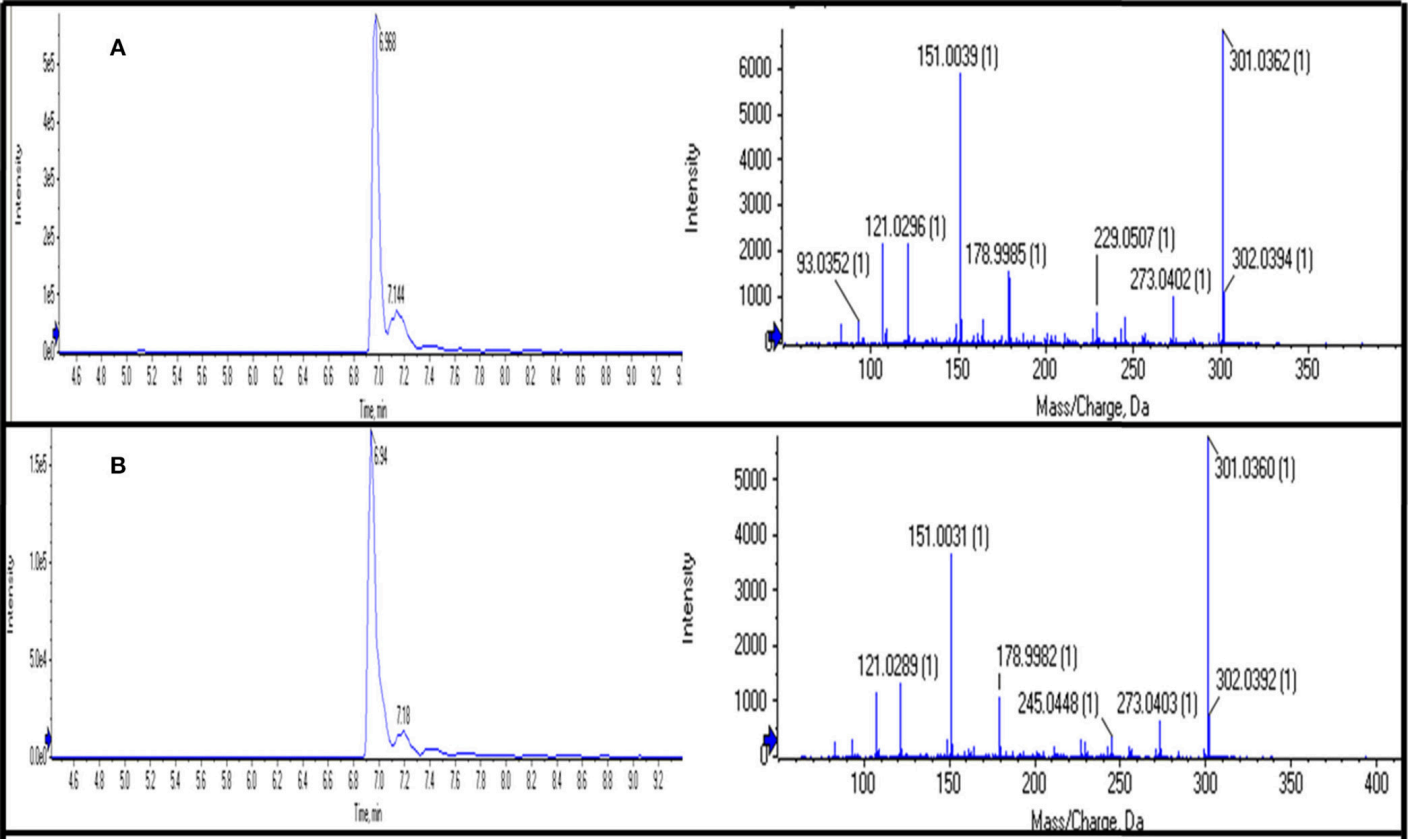
**Chromatograms of UPLC-QTOF-MS/MS showing retention time (RT) and MS/MS fragmentation of “Quercetin” using its standard (A)** and in sample **(B)**.

**Figure 4:**
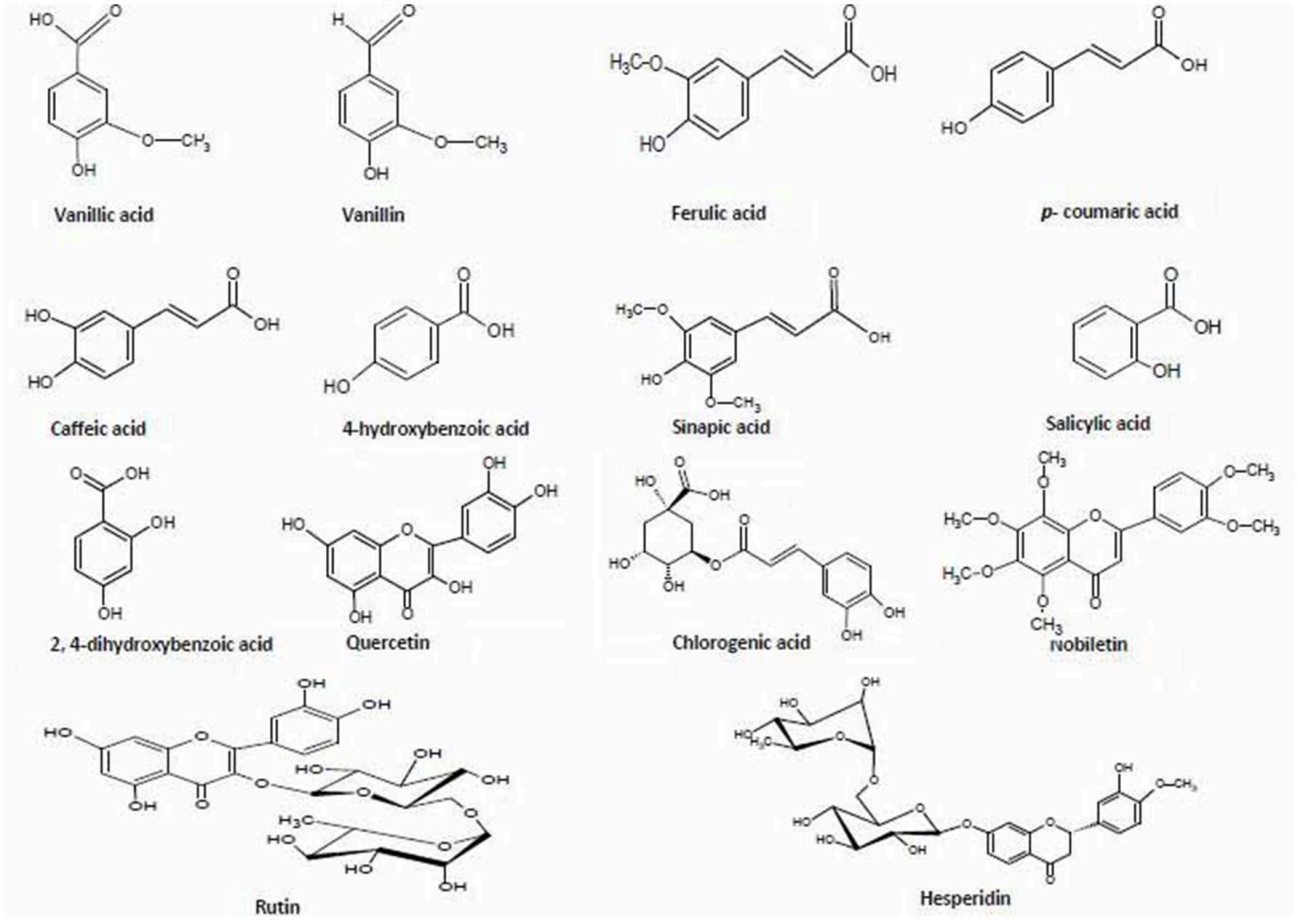
**Chemical structures of 14 phenolic compounds used for validation and quantification in the six extracts of two diverse bread wheat (*Triticum aestivum*) varieties, “C306” and “Sonalika,” differing for chapatti (unleavened flat bread) quality**.

Out of 69 PCs, the standards of 17 PCs were randomly selected for their validation on UPLC-QTOF-MS/MS. Fourteen PCs (14/17~82%) were validated by matching their RT, MS, and MS/MS (Figure 3 and Supplementary Table 2), while the remaining three PCs such as *t*-cinnamic acid, benzoic acid, and astringin were not validated. The precursor ion and MS/MS fragmentation patterns of the fourteen compounds (Table 2) were as: vanillic acid (167; 152, 108, 123), vanillin (151; 136, 108, 92), ferulic acid (193; 134, 178), *p*-coumaric acid (163; 119, 93), caffeic acid (179; 135, 89), quercetin (301; 273, 178, 151), rutin (609; 301, 271), salicylic acid (137; 93), chlorogenic acid (353; 263, 190, 146, 102), hesperidin (611; 303, 465), 2,4-dihydroxybenzoic acid (153; 108), nobiletin (401; 341, 189), sinapic acid (223; 208, 193, 149, 121), and *4*-hydroxybenzoic acid (139; 121, 99, 75). Their structural formula was represented in Figure 4.

**Table 2:** **Validation of the tentatively identified phenolic compounds using their standards on UPLC-QTOF-MS/MS**.

**Phenolic compound**	**Exact mass**	**^a^Expt. mass**	**Slope**	**Intercept**	**^b^*R*^2^**	**^c^LOD (ng/ml)**	**^d^LOQ (ng/ml)**	**Precursor ion mass**	**^e^MS/MS**
Vanillic acid	168.0422	167.0422	8.377	−170.350	0.9614	0.3	0.4	167	167, 152, 123, 108
Vanillin	152.0473	151.0473	53.105	−19.293	0.9985	0.2	0.4	151	151, 136, 108, 92
Ferulic acid	194.0579	193.0579	113.846	2442.610	0.9349	0.3	0.4	193	193, 178, 134
*p*-Coumaric acid	164.0473	163.0473	244.990	859.603	0.9956	0.3	0.4	163	163, 119, 93
Caffeic acid	180.0422	179.0422	1120.833	−2045.589	0.9996	0.2	0.4	179	179, 135, 89
Quercetin	302.0426	301.0426	1052.024	3.418	0.9892	0.3	0.4	301	301, 273, 178, 151
Rutin	610.1533	609.1533	819.436	2068.880	0.9960	0.3	0.4	609	609, 301, 271
Salicylic acid	138.0316	137.0316	96.887	8378.975	0.9992	0.1	0.3	137	137, 93
Chlorogenic acid	354.0950	353.0950	550.314	1604.424	0.9983	0.2	0.4	353	353, 263, 190, 146, 102
2,4-dihydroxybenzoic acid	154.0266	153.0266	86.953	−511.931	0.9982	0.2	0.3	153	153, 108
Nobiletin	402.1314	401.1314	13.610	−48.786	0.9995	0.6	1.0	401	401, 341, 189
Hesperidin^*^	610.1897	611.1897	1455.044	553.506	0.9948	0.6	0.8	611	611, 465, 303
Sinapic acid^*^	224.0684	225.0684	37.385	−35.751	0.9974	0.6	0.8	223	223, 208, 193, 149, 121
*p*-hydroxybenzoic acid^*^	138.0316	139.0316	13.630	49.741	0.9982	0.5	1.0	139	139, 121, 99, 75

a Experimental mass that was obtained after addition or deletion of H^+^ or H^−^ ion;

b R^2^, coefficient of determination obtained through regression analysis;

c LOD, Limit of detection;

d LOQ, Limit of quantitation;

e MS/MS, mass of the ions produced from fragmentation of precursor ion; Phenolic compounds were detected in negative mode of ionization except the compounds marked with

“*” *in positive mode of ionization*.

The identification and characterization of PCs was accomplished using ICH guidelines (ICH, 2005). The correlation coefficient (*R*^2^) between different concentration of each standard and their respective peak areas was >0.99 except for ferulic acid (0.93), quercetin (0.98), and vanillic acid (0.96) (Table 2). This also shows that the method developed has good linearity (Table 2). LOD and LOQ values of standards were in the range of 0.1–0.6 ng/ml (lowest for salicylic acid) and 0.3–1.0 ng/ml (lowest for salicylic acid and 2,4-dihydroxybenzoic acid), respectively. Except hesperidin, sinapic acid, and *p*-hydroxybenzoic acid, the fragmentation pattern, LOD and LOQ of eleven phenolic compounds was carried out in the −ve mode because their respective peak was sharper and their intensity was high in −ve mode as compared to the +ve mode. LOD and LOQ of PCs were very low (within 1 ng/ml) and better than the earlier reported in wheat (>50 ng/ml; Nicoletti et al., 2013). Intra-day variation (Relative standard deviation, RSD) in retention time (RT) for 14 PCs ranged from 0.09 to 2.18% and that for Inter-day from 0.32 to 3.27% (Table 3). For peak area, Intra-day variation (%RSD) for 14 PCs ranged from 0.22 to 2.2% and that for Inter-day from 1.16 to 4.13% (Table 3).

**Table 3:** **Precision (%) (Relative standard deviation in percentage) of phenolic compounds**.

**Phenolic compound**	**Concentration (ng/ml)**	**Retention time (min)**		**Peak area**	
		**^a^Intra-day**	**^b^Inter-day**	**Intra-day**	**Inter-day**
Vanillic acid	50	0.71	1.21	0.22	1.16
	500	0.61	0.89	1.42	3.46
	1000	0.88	2.14	1.61	4.13
Vanillin	50	0.42	1.04	1.46	2.71
	500	2.18	2.39	1.72	4.02
	1000	2.08	3.06	1.74	2.93
Ferulic acid	50	0.97	1.49	2.07	4.11
	500	0.97	1.99	1.91	3.13
	1000	1.07	1.89	0.8	2.81
*P*-Coumaric acid	50	0.63	1.54	1.04	2.19
	500	1.58	1.72	0.54	2.87
	1000	0.52	0.65	1.09	3.22
Caffeic acid	50	0.71	0.98	1.24	3.25
	500	0.71	0.86	0.58	2.92
	1000	0.71	2.06	1.83	2.91
Quercetin	50	0.82	0.99	0.67	2.79
	500	0.78	1	0.98	1.74
	1000	0.15	0.66	1.96	2.79
Rutin	50	0.2	1.3	0.74	3.26
	500	0.2	1.45	1.24	3.24
	1000	0.2	1.47	0.9	3.3
Salicylic acid	50	0.09	1.1	0.92	2.31
	500	0.97	1.77	0.81	3.18
	1000	0.7	1.45	1.64	3.84
Chlorogenic acid	50	0.36	1.25	0.87	3.23
	500	0.36	1.12	1.74	3.72
	1000	0.36	2.15	1.08	3.57
2,4-dihydroxybenzoic acid	50	1.39	3.27	1.57	2.1
	500	1.46	1.78	1.38	3.27
	1000	1.18	1.7	1.84	3.61
*p*-Hydroxy benzoic acid	50	0.09	0.43	1.35	1.99
	500	0.17	0.32	1.56	3.44
	1000	0.7	0.81	0.99	2.82
Hesperidin	50	0.98	2.45	1.93	3.41
	500	0.87	2.13	1.7	4.09
	1000	1.06	1.37	1.52	3.62
Sinapic acid	50	0.37	1.77	0.5	2.65
	500	0.1	0.88	0.91	3.1
	1000	0.27	0.54	2.2	3.26
Nobilitin	50	1.69	3.01	1.96	3.23
	500	0.51	1.67	0.26	2.15
	1000	1.71	2.02	1.4	3.58

a Intraday, data were taken in triplicates at three different intervals within the same day;

b *Interday, data were taken in triplicates at 3 consecutive days*.

### Quantitative measurement of phenolic compounds in good and poor chapatti (unleavened flat bread) quality bread wheat varieties

Quantification of the 14 phenolic compounds in the six wheat samples (free, alkaline, and acidic extracts) of two diverse bread wheat varieties, “C306” and “Sonalika” differing for chapatti quality was done on UPLC-QTOF-MS (Table 4). These PCs were highly concentrated in acidic extracts as compared to the free and alkaline extracts. In the poor chapatti quality bread wheat variety, Sonalika, the concentration of the fourteen PCs was higher as compared to that of the good chapatti quality variety, “C306.” Among 14 PCs, ferulic acid was found to be highly concentrated in acidic extract in grains of the variety, “Sonalika” (232.64 ± 2.24 μg/100 g; Table 4). In this variety six PCs such as ferulic acid, salicylic acid, vanillic acid, quercetin, rutin, and vanillin were present in at least 1 ppm (>100 μg/100 g) in grain. Quercetin was not detected in the good chapatti quality variety, “C306.”

**Table 4:** **Quantification (μg/100 g, mean ± *SD*) of phenolic compounds in three extracts (free, alkaline, and acidic) of the two diverse bread wheat varieties, “C 306” and “Sonalika” differing for chapatti (unleavened flat bread) quality**.

**Phenolic compounds**	**C306**			**Sonalika**		
	**^#^Free**	**^#^Acidic**	**^#^Alkaline**	**^#^Free**	**^#^Acidic**	**^#^Alkaline**
4-hydroxybenzoic acid	2.76^a^ ± 0.09	8.31^b^ ± 0.12	2.87^b^ ± 0.07	24.57^c^ ± 0.15	79.12^e^ ± 1.08	11.13^d^ ± 0.01
Vanillic acid	7.54^a^ ± 0.07	23.43^c^ ± 0.14	0.86^b^ ± 0.09	83.56^d^ ± 0.44	169.21^f^ ± 0.09	39.25^e^ ± 0.71
Vanillin	2.29^a^ ± 0.09	5.98^c^ ± 0.11	0.82^b^ ± 0.02	22.33^d^ ± 0.62	104.88^f^ ± 0.91	11.07^e^ ± 0.08
Ferulic acid	1.85^a^ ± 0.00	12.64^c^ ± 0.07	*N*.*D*	28.42^d^ ± 0.67	232.64^f^ ± 2.24	26.23^e^ ± 0.17
*p*-Coumaric acid	1.42^a^ ± 0.02	6.02^c^ ± 0.03	0.30^b^ ± 0.01	15.60^d^ ± 0.14	72.48^e^ ± 0.03	15.47^d^ ± 0.09
Caffeic acid	2.02^a^ ± 0.02	05.43^c^ ± 0.03	1.20^b^ ± 0.00	18.10^d^ ± 0.42	46.88^f^ ± 0.25	9.63^e^ ± 0.38
Quercetin	N.D	N.D	N.D	22.97^b^ ± 0.89	116.57^d^ ± 0.29	6.87^c^ ± 0.71
Rutin	1.53^a^ ± 0.10	3.85^c^ ± 0.17	0.49^b^ ± 0.14	20.62^d^ ± 1.24	105.58^f^ ± 0.49	9.64^e^ ± 0.42
Salicylic acid	3.24^a^ ± 0.19	13.78^c^ ± 0.69	1.02^b^ ± 1.10	60.11^d^ ± 2.00	195.15^f^ ± 1.01	28.59^e^ ± 0.57
Chlorogenic acid	1.17^a^ ± 0.07	4.82^a^ ± 0.17	0.03^b^ ± 0.01	17.01^c^ ± 0.47	85.86^d^ ± 1.69	8.89^e^ ± 0.21
2,4-dihydroxybenzoic acid	1.85^a^ ± 0.06	3.42^b^ ± 0.34	1.43^a^ ± 0.03	13.67^c^ ± 0.54	84.88^e^ ± 1.48	6.83^d^ ± 0.20
Hesperidin	0.16^a^ ± 0.03	0.29^b, c^ ± 0.11	0.05^a, b^ ± 0.02	00.80^c^ ± 0.03	2.89^e^ ± 0.21	00.39^d^ ± 0.01
Nobiletin	1.87^a^ ± 0.11	2.88^b^ ± 0.11	1.47^a^ ± 0.06	10.28^c^ ± 0.21	51.59^e^ ± 0.50	6.93^d^ ± 0.12
Sinapic acid	1.73^a^ ± 0.11	6.29^b^ ± 0.14	1.76^a^ ± 0.07	16.30^c^ ± 0.22	67.68^e^ ± 0.55	8.09^d^ ± 0.13

N.D, not detected;

# *concentration in (μg/100 g). The letters “a–f” next to the average values are based on Duncan mean value test. The mean values with same letter are not statistically significantly different (p < 0.05), whereas those with different letter are statistically significantly different*.

### Expression of phenolic compound biosynthesis pathway genes in good and poor chapatti (unleavened flat bread) quality bread wheat varieties using wheat microarray data

**Figure 5:**
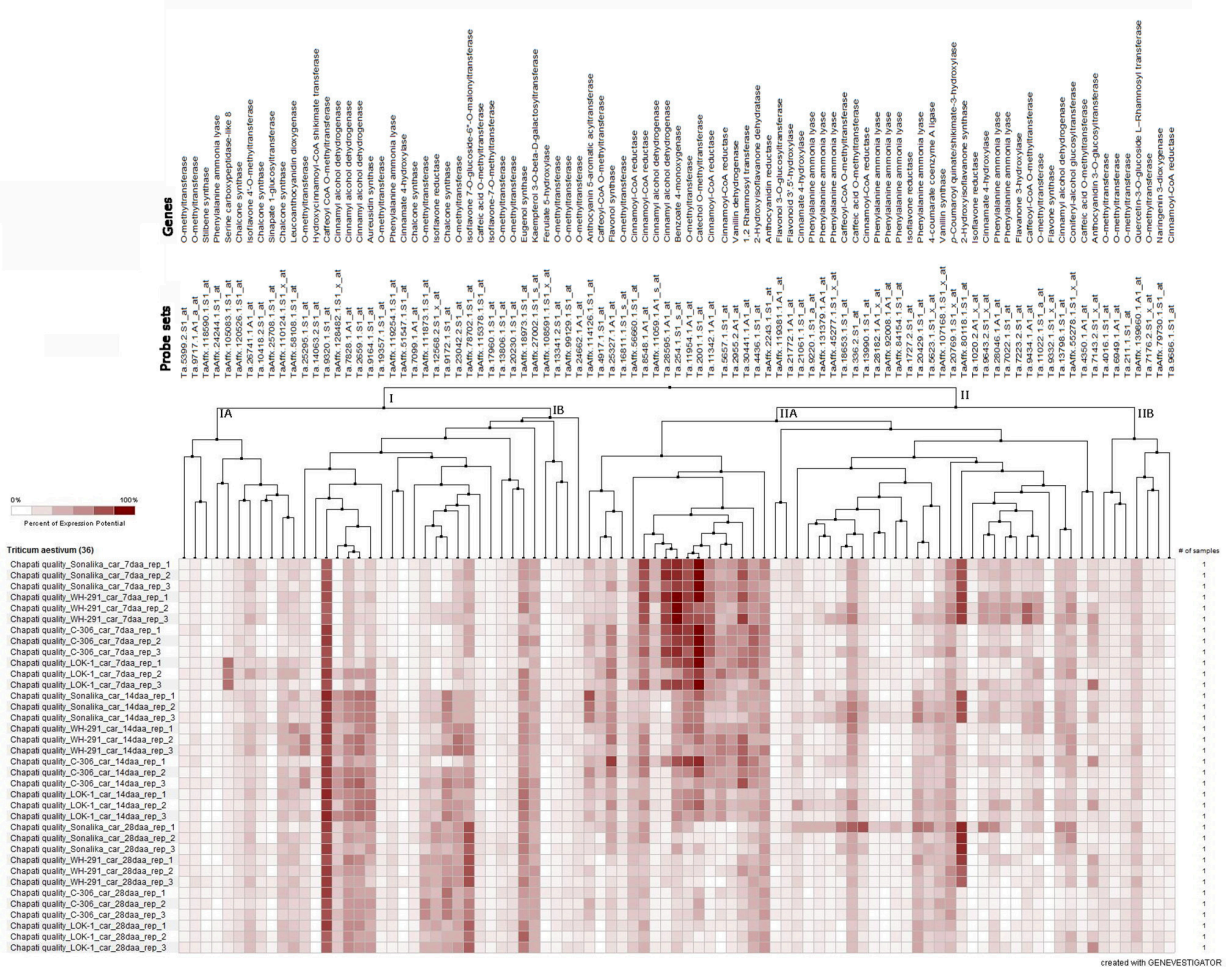
**Heat map of the expression level of 91 probe sets of 41 phenolic compound biosynthesis pathway genes at three seed developmental stages (7, 14, and 28 days after anthesis DAA) in three replications (Rep_1, Rep_2, and Rep_3) in the two good chapatti (unleavened flat bread) bread wheat varieties, “C 306” and “Lok1” and two poor chapatti varieties, “Sonalika” and “WH 291.”** The expression data of the genes were extracted from the microarray data of Singh et al. (2014). The heat map was generated using Genevestigator software (https://genevestigator.com/).

The microarray expression data (Affymetrix's GeneChip® Wheat Genome Arrays, Singh et al., 2014) of 91 probe sets for 41 genes related to phenolic compound biosynthesis pathway revealed the temporal distribution pattern of the majority of the probe sets during seed development in the wheat varieties (Figure 5). The expression of about 51% of the probe sets was very low and/or not detected and that of the remaining probe sets were either in early stage or late stage of seed development. The expression level of the probe set, Ta.9320.1.S1_at of caffeoyl CoA O-methyl transferase was high during seed development in all the varieties. The probe set, TaAffx_80118.1.S1_at of *2*-hydroxy isoflavanone synthase highly expressed in the poor chapatti quality varieties (“Sonalika” and “WH 291”) during seed development. The five probe sets, namely Ta.8548.1.A1_at, Ta.26595.1.A1_at, Ta.254.1.S1_s_at, Ta.11954.1.A1_at, and Ta.2001.1.S1_at of cinnamoyl-CoA reductase, cinnamoyl alcohol dehydrogenase, benzoate 4-monoxygenase, O-methyl transferase, and catechol O-methyl transferase, respectively, showed strong expression potential in early stage of seed development in the varieties (Figure 5). On the basis of expression potential, the ninety-one probe sets were divided into two groups-I comprising of 37 probe sets and II of 54 probe sets. The expression of the majority of the probe sets in group-IA and IIB showed low expression level (Figure 5).

The fold change analysis showed that out of 91, 44 probe sets representing 23 genes showed ≥1.5-fold change difference in expression between the good (“C 306”) and poor (“Sonalika”) chapatti varieties (Table 5). About 45, 64, and 73% of the probe sets showed the negative expression in the good chapatti variety, “C 306” at early (7 DAA), mid (14 DAA), and late (28 DAA) stage of seed development, respectively. The probe set, TaAffx_80118.1.S1_at of *2*-hydroxy isoflavanone synthase showed highly negative expression throughout seed development in the good chapatti quality variety, “C 306.” The four probe sets, Ta.9643.2.S1_x_at, Ta.13798.1.S1_at, Ta.13990.1.S1_at, and TaAffx_80118.1.S1_at, of cinnamate 4-hydroxylase, cinnamyl alcohol dehydrogenase, cinnamoyl CoA reductase, and *2*-hydroxy isoflavanone synthase, respectively showed about five-fold negative expression at late stage of seed development in the good chapatti quality variety, “C 306.” The two probe sets, Ta.9172.1.S1_at and Ta.2001.1.S1_at, of chalcone synthase and catechol O-methyl transferase, respectively showed at least four-fold positive expression at late stage of seed development in the good chapatti variety, “C 306.” The chromosome location of the 44 probe sets was determined by homology search with the IWGSC's wheat whole genome sequence databases (Table 5). The chromosome location revealed that the majority of 23 genes were coded by multiple loci.

**Table 5:** **Differential expression level (at least 1.5-fold change) of phenolic compound biosynthesis pathway genes between two diverse bread wheat varieties, “C306” and “Sonalika,” differing for chapatti (unleavened flat bread) quality during seed development (7, 14, and 28 days after anthesis, DAA)**.

**Gene/Probe set**	Chr^*^	**Differential gene expression level (Fold change)**		
		**7 DAA**	**14 DAA**	**28 DAA**
**PHENYLALANINE AMMONIA LYASE**				
Ta.20429.1.S1_at	2 BL	1.2	−3.9	−2.1
Ta.28046.1.A1_at	2 BL	−1.2	−2.8	−3.9
Ta.28182.1.A1_x_at	1 DS	1.0	−1.6	−1.9
Ta.7022.1.S1_at	2 DS	−1.7	−1.7	−1.9
Ta.9220.1.S1_a_at	4 AL	1.1	−1.5	−2.8
TaAffx.131379.1.A1_at	2 DS	1.1	−2.4	−2.1
TaAffx.45277.1.S1_x_at	2 AS	1.4	−1.8	−2.3
TaAffx.92008.1.A1_at	2 AL	−1.1	−2.0	−2.3
**CINNAMATE 4-HYDROXYLASE**				
Ta.9643.2.S1_x_at	3B	−1.2	−1.8	−4.6
Ta.21061.1.S1_at	7AL	1.2	1.2	−2.0
**4-COUMARATE CoA LIGASE**				
Ta.5623.1.S1_x_at	6 AS	1.7	−1.3	−2.2
**CINNAMYL ALCOHOL DEHYDROGENASE**				
Ta.13798.1.S1_at	6 AS	1.2	−1.9	−5.0
Ta.2659.1.S1_at	5 BL	−1.2	−1.3	1.6
Ta.28595.1.A1_at	7 BL	−1.2	2.0	1.1
**CINNAMOYL-CoA REDUCTASE**				
Ta.11342.1.A1_at	6 BS	1.8	1.6	−1.0
Ta.5657.1.S1_at	3 AL	−1.0	1.6	1.3
Ta.8548.1.A1_at	7 DL	−1.5	3.3	−1.8
Ta.13990.1.S1_at	5 AL	−1.2	−3.3	−6.3
**CAFFEIC ACID O-METHYLTRANSFERASE**				
Ta.336.2.S1_at	7 DL	−1.3	−4.7	−3.8
Ta.4350.1.A1_at	3 DL	1.0	1.0	−1.5
**CAFFEOYL-CoA O-METHYLTRANSFERASE**				
Ta.18653.1.S1_at	7 DS	1.0	−2.8	−3.3
Ta.4917.1.S1_at	7 AS	1.0	−1.2	−1.7
Ta.9434.1.A1_at	7 AS	1.0	−2.1	−1.2
**BENZOATE 4-MONOXYGENASE**				
Ta.254.1.S1_s_at	6 AL	1.0	1.7	−2.4
**VANILLIN DEHYDROGENASE**				
Ta.2955.2.A1_at	7 DL	1.2	1.8	1.4
**O-METHYLTRANSFERASE**				
Ta.23042.2.S1_at	2 DS	−1.4	−1.0	−1.6
Ta.25295.1.S1_at	2 DS	−1.1	3.3	1.2
TaAffx.111873.1.S1_at	7 DS	−1.1	1.1	2.8
Ta.11022.1.S1_a_at	6 AS	−2.3	−2.0	−2.5
**CONIFERYL-ALCOHOL GLUCOSYLTRANSFERASE**				
TaAffx.55278.1.S1_x_at	2 BL	1.0	1.1	−3.0
**CHALCONE SYNTHASE**				
Ta.9172.1.S1_at	2 BL	1.1	−5.5	7.1
**FLAVANONE 3-HYDROXYLASE**				
Ta.7223.2.S1_at	4 BL	−1.4	−1.9	−1.5
**FLAVONOL SYNTHASE**				
Ta.25327.1.A1_at	6 BL	2.8	2.0	−2.5
**ISOFLAVONE 7-O-GLUCOSIDE-6”-O-MALONYLTRANSFERASE**				
TaAffx.78702.1.S1_at	5 BS	1.3	1.4	−2.1
**ISOFLAVONE REDUCTASE**				
Ta.1020.2.A1_x_at	1 BL	−1.8	−1.2	−1.7
Ta.1727.2.S1_at	2 DS	−1.1	−1.5	−1.3
Ta.12568.2.S1_x_at	7 BS	1.0	−1.4	−1.5
**CATECHOL O-METHYLTRANSFERASE**				
Ta.2001.1.S1_at	3 DS	−1.1	1.7	4.1
**1,2 RHAMNOSYL TRANSFERASE**				
Ta.30441.1.A1_at	2 BS	−1.5	2.5	1.3
**2-HYDROXYISOFLAVANONE DEHYDRATASE**				
Ta.4436.1.S1_at	4 AS	1.7	1.4	1.3
**2-HYDROXYISOFLAVANONE SYNTHASE**				
TaAffx.80118.1.S1_at	3 DS	−19.4	−30.5	−48.5
**ANTHOCYANIDIN 3-O-GLUCOSYLTRANSFERASE**				
Ta.7143.2.S1_x_at	1 AS	2.6	−1.3	1.4
**ANTHOCYANIN 5-AROMATIC ACYLTRANSFERASE**				
TaAffx.114126.1.S1_at	7 AL	1.2	−1.7	1.5
**AUREUSIDIN SYNTHASE**				
Ta.9164.1.S1_at	5 BL	1.4	−2.5	−1.2

* *Chr, chromosome*.

*The gene expression data was produced on the wheat microarrays (Singh et al., 2014)*.

### Expression of phenolic compound biosynthesis pathway genes in good and poor chapatti (unleavened flat bread) quality bread wheat varieties using qRT-PCR

Real-time expression analysis of seventeen genes related to phenolic compound biosynthesis pathway revealed differential expression during seed developmental stages between the two diverse bread wheat varieties, “C 306” and “Sonalika” differing for chapatti quality (Table 6; Supplementary Table 3; Supplementary Material 1). Eleven genes showed low expression in the good chapatti variety, “C 306” in comparison to the poor chapatti variety, “Sonalika” at mid (14 DAA) and late stage (28 DAA) of seed development (Table 6). One gene, phenylalanine ammonia lyase-5 (PAL-5), showed high expression in the good chapatti variety during seed development. Three genes, *4*-coumarate CoA ligase (4-CL), chalcone synthase (CHS), and caffeic acid-O-methyl transferase (COMT) showed low expression at mid stage and high expression in the late stage (28 DAA) of seed development in the good chapatti variety. Two genes, ferulate-5-hydroxylase-1 (F5H-1) and caffeoyl CoA O-methyl transferase (CCoMT-1), showed high expression at mid stage and low expression at the late stage of seed development.

**Table 6:** **Detailed information of primers for 17 phenolic compound biosynthesis pathway genes for real-time quantitative gene expression analysis (qRT-PCR)**.

**Gene**	**Probe set ID^a^**	**Chr^b^**	Primer^*^	**Sequence (5′-3′)**	**Tm (°C)**	**Amplicon size (bp)**
Phenylalanine ammonia lyase (PAL 5)	Ta.28046.1.A1_at	2BL	F	AGGAGCGCCGTGGAGAATGGCACT	625	141
			R	CTCTTCGCCAGGAGACCGCGTCTT	625	
Cinnamate 4-hydroxylase (C4H1)	Ta.9643.2.S1_x_at	3B	F	CATCCCGCTGCTGGTGCCCCA	625	123
			R	TCCACTGCTCCGGGTTGTTGGCGA	625	
*4*-Coumarate coenzyme A ligase (4CL)	Ta.5623.1.S1_x_at	6AS	F	CTACAAGAGGATCCACAAGGT	524	159
			R	TGCTGGCTGGCTGAGTGCCTG	602	
*p*-coumaroyl quinate/shikimate-3-hydroxylase (C3′H)	Ta.20769.1.S1_x_at	1AL	F	TCGCTGCTCCCGTTCGCCATC	602	134
			R	GGCTTGATCTGCCGCAGGTTG	583	
Caffeoyl-CoA O-methyltransferase 1 (CCoMT1)	Ta.18653.1.S1_at	4AL	F	TGCTGATCAAGATGGCGGGCG	583	129
			R	ACTCGCGGTCGGTGTCGATGG	602	
Cinnamoyl-CoA reductase (CCR)	Ta.13990.1.S1_at	5BL	F	TATACGAGACGGTGAAGAGCCTCC	591	180
			R	GTCCCATAGAATTAACTAGCCGACAGC	597	
Cinnamyl alcohol dehydrogenase (CAD)	Ta.13798.1.S1_at	6AS	F	GGTGCTCCAGTTCTGCGTCGACAA	608	157
			R	TCAGGCGGCGTCCTCGATGTT	583	
Ferulate 5-hydroxylase 1 (F5H1)	Ta.Affx108591.1.S1_x_at	1BL	F	ACACCGTCGAATCGAACACCCAAG	591	147
			R	CGAAGACCACCGAGGCGACGAACA	625	
Caffeic acid -O-methyl transferase (COMT)	Ta.336.2.S1_at	7DL	F	AAGAACGCCATCGAGCTGGGTCTC	608	180
			R	CTCCTCCATCGTGCACGACACCAC	625	
Chalcone synthase (CHS)	Ta.9172.1.S1_at	2AL	F	CTTCCACCTGCTCAAGGACGTGCC	625	267
			R	GAGCGCTTGCGCATCTCATCCATG	608	
Flavonol synthase1 (FLS1)	Ta.25327.1.A1_at	6AL	F	GGTGTTCAAGGACGGATGCTGGTA	591	157
			R	CGGCCACGACATCCGCGTCTT	602	
Flavone synthase (FNS)	Ta.9332.1.S1_x_at	5DL	F	TGATGGGCAAGAAGTACCTGGAGA	574	166
			R	TACCCCTGCAGGTCCATCCAG	583	
1,2 Rhamnosyl transferase (1,2 RhaT)	Ta.30441.1.A1_at	2BS	F	CGGCCGTTCTTCGTCGTGCTCAA	606	134
			R	GCTGCTGCACCCACCCCGTGT	622	
Benzoate 4-monoxygenase (B4MO)	Ta.254.1.S1_s_at	6DL	F	CGCCGAGGACATGCACGAGTG	602	199
			R	GAAGACGCCGTGCCAGAAGGG	602	
Quercetin-3-O-glucoside L–Rhamnosyl transferase (Q3-GRhaT)	Ta.Affx139860.1.A1_at	3B	F	GGCACGCGGGATGGTGGTTTC	602	142
			R	GGTCCAGCTCAGCATCGTCAC	583	
Vanillin dehydrogenase (VDH)	Ta.2955.2.A1_at	7DL	F	ACTACATCGAGCCCACGGTTTTCA	574	140
			R	GTACCTCGTGTCGTTGGCCCT	642	
Vanillin synthase (VS)	Ta.Affx107168.1.S1_x_at	5BL	F	CTTCCATCCCAGGCATTTGAGTAC	574	216
			R	GATCACCTCAAAGGCAACACTCAC	574	

a The primer pairs were designed using the probe sets of GeneChip®; Wheat Genome Array (Affymetrix, Santa Clara, USA) corresponding to eighteen phenylpropanoid pathway genes.

b Chromosome (Chr) mapping was done using IWGSC's wheat genome sequences;

* *F, forward and R, reverse primer*.

## Discussion

### Characterization of phenolic compounds in good and poor chapatti (unleavened flat bread) quality bread wheat varieties

Phenolic compounds are ubiquitous in plant and they represent a large group of biomolecules. PCs have both structural and functional properties. The role of PCs in biotic and abiotic stresses has been extensively studied in plants. Majority of PCs possess health benefits due to their unique structure features. However, their role in processing quality is limited in cereal crops including wheat. The effect of PCs on bread quality has been studied and they play largely negative role. It is presumed that they can also affect other type of end-use food products such as chapatti which is unleavened flat bread. Chapatti is now globally recognized an end-use wheat food product. The characterization of PCs using modern analytical tools such as UPLC-QTOF-MS or MS/MS and functional genomics analysis of their biosynthesis genes in diverse bread wheat varieties differing for chapatti quality have not been studied. In this study, identification and quantifcation of PCs and functional genomics of their biosynthesis genes and their comparative analysis have been studied in a good chapatti variety and a gold standard variety, “C 306” and a poor chapatti variety, “Sonalika.” In this study, a very large number (69) of PCs were identified in their seeds. Dinelli et al. (2009, 2011) had identified ≥70 PCs using a small set of wheat germplasm. Most of the PCs were detected in bound forms (acidic and alkaline extract), which were in agreement with the previous studies (Dinelli et al., 2009, 2011; Vaher et al., 2010 and Nicoletti et al., 2013). In the good chapatti variety, “C 306,” nine PCs (hinokinin, coutaric acid, fertaric acid, *p*-coumaroylqunic acid, kaempferide, isorhamnetin, epigallocatechin gallate, methyl isoorientin-2′-O-rhamnoside, and cyanidin-3-rutinoside) were identified and these were not identified in the poor chapatti variety, “Sonalika” in this study. Similarly, four PCs (tricin, apigenindin, quercetin-3-O-glucuronide, and myricetin-3-glucoside) were only identified in the poor chapatti variety, “Sonalika.” Therefore, about 20% (9+4 = 13 PCs) of the identified PCs are unique to each other and may be “variety or genotype” specific PCs. They may be informative after further validation.

The concentration of PCs in wheat determined in this study is in the range of (0.03 ± 0.01 μg/100 g) for chlorogenic and *p*-coumaric acid to (232.64 ± 2.24 μg/100 g) for ferulic acid and is very low in comparison to medicinal herbs as in our study they are present in microgram per 100 g samples where as in medicinal plants they are present in milligram per 100 gram of samples as reported by Chen et al. (2012). However, the content of PCs in wheat food products reported by Vaher et al. (2010) and Nicoletti et al. (2013) was several folds higher than our study except few PCs. This may be due to environmental effect on the wheat plants taken for study and also due to the genotype of the plant. Both these factors affect the phenolic content (Mpofu et al., 2006). Also phenolic content is affected by method of extraction (Verma et al., 2009). Of these 69 PCs, 14 PCs were validated using their standards (Table 2). Retention time (RT) and fragmentation pattern of the PCs were matched with that of their standards. The fragmentation pattern of ferulic acid (193, 178) and caffeic acid (179, 135) were similar to that reported by Wang et al. (2012) and that of vanillic acid (167, 152, 123, 108), sinapic acid (223, 208, 193, 149, 121), chlorogenic acid (353, 191) were similar to that reported by Theerasin and Baker (2009). The concentration of all 14 PCs was found to be higher in acidic extract of “Sonalika.” The concentration of hesperidin was found to be lowest among 14 PCs in all the three extracts of “C 306” and “Sonalika.” Quercetin was present only in three extracts of “Sonalika” while it was absent in “C 306.” Similarly, ferulic acid was absent in alkaline extract of “C 306” while present in remaining extracts of “C 306” and “Sonalika.” It is present in high concentration (232.64 ± 2.24 μg/100 g) in acidic extract of Sonalika which is in agreement with previous studies which shows ferulic acid is most abundant phenolic compound in wheat grain (Moore et al., 2006 and Zilic et al., 2012b). Among plant metabolites, phenolic compounds are most profuse, and ubiquitous having antioxidant activities which positively affect human health (Kim et al., 2006 and Li et al., 2008). Ferulic acid in alkylated form is a potent anti-carcinogen (Imaida et al., 1990) and also exhibit anti-apoptotic effect on peripheral blood mononuclear cells (PBMCS) exposed to H_2_O_2_ induced oxidative stress (Khanduja et al., 2006). Similarly, caffeic acid and its derivatives act as inhibitor of HIV-1 integrase (Bailly and Cotelle, 2005; Bailly et al., 2005), asthma and allergic reactions (Koshihara et al., 1984). Quercetin is a versatile flavonol with several pharmacological functions such as antioxidant, neurological, antiviral, anticancer, cardiovascular, antimicrobial, anti-inflammatory, hepatoprotective, protective of the reproductive system, and anti-obesity agent (Jan et al., 2010; Dajas, 2012; Joseph and Muralidhara, 2013). Similarly other flavonoids and phenolic acids identified in this study- hesperidin, nobiletin, rutin, vanillic acid, vanillin, sinapic acid, etc. have several health benefits. In this study, all 14 PCs showed high variation among different extracts and between the varieties. Therefore, PCs can be extended to a larger wheat germplasm set for determination of their genetics factors or DNA-based molecular markers using QTL and/or association mapping approaches (Roy et al., 1999, 2010).

### Expression of phenolic compound biosynthesis pathway genes in good and poor chapatti (unleavened flat bread) quality bread wheat varieties

Phenolic compounds identified in this study are biosynthesized via phenylpropanoid biosynthesis pathway. In this pathway, PAL is the key enzyme as it converts the amino acid, phenylalanine, into *t*-cinnamic acid (Nair et al., 2004). *t*-cinnamic acid is converted into *p*-coumaric acid by cinnamate-4-hydroxylase (C4H) (Boerjan et al., 2003) and *p*-coumaric acid is further converted to produce several phenolic acids and flavonoids by a series of enzymes (Figure 1). The genes of phenolic compound biosynthesis pathway enzymes were analyzed for their expression level in the two diverse bread wheat varieties differing for chapatti (unleavened flat bread) using microarray and real-time quantitative PCR (qRT-PCR). In order to study the expression pattern of seventeen phenylpropanoid pathway biosynthesis genes in the two varieties, qRT-PCR was done at two seed developmental stages i.e., 14 DAA and 28 DAA (Table 7). Out of 17, 12 genes were phenolic acid biosynthesizing genes including PAL, vanillin dehydrogenase, vanillin synthase, caffeic acid-O-methyl transferase, caffeoyl CoA O-methyl transferase, hydroxy cinnamoyl-CoA shikimate transferase, 4-hydroxycinnamoyl CoA ligase, ferulate 5 hydroxylase 1, cinnamoyl-CoA reductase, cinnamyl alcohol dehydrogenase, cinnamate-4-hydroxylase 1, *p*-coumaroyl quinate/shikimate-3-hydroxylase, benzoate-4-monoxygenate and the remaining five genes were flavonoid biosynthesis pathway genes including chalcone synthase, flavone synthase, flavonol synthase, quercetin- 3-O-glucoside L–rhamnosyl transferase, and 1,2-rhamnosyl transferase. At mid stage (14 DAA) of seed development, the expression of 15 genes (83%) was higher whereas at 28 DAA stage, expression of 14 genes (77%) was higher in the poor chapatti variety, “Sonalika,” in comparison to the good quality variety “C 306.” However, about 50% (8 out of 17 genes) of gene expression data of microarrays and qRT-PCR are in agreement i.e., validated at least at the late stage (28 DAA) of seed development. Among them, only chalcone synthase showed high expression in the good chapatti variety and the remaining seven genes showed low expression in the good chapatti variety. Chalcone synthase is a key enzyme in flavonoid/isoflavonoids biosynthesis pathway and catalyses the conversion of *p*-coumaroyl CoA into naringenin chalcone (Dao et al., 2011; Ma et al., 2016). The seven genes are flavonol synthase, cinnamyl alcohol dehydrogenase, cinnamate 4-hydroxylase, benzoate 4-monoxygenase, caffeoyl- CoA O-methyl transferase, quercetin-3-O-glucoside L-rhamnosyl transferase, and flavone synthase. These genes are responsible for biosynthesis of key enzymes of hydroxy-cinnamic acid, hydoxy-benzoic acid, and flavonoid pathways. Hence the majority of phenolic compounds showed low concentration in good chapatti variety may be collaborated with that of low expression of the seven genes which are identified both through the microarray and qRT-PCR data. These eight genes are candidate genes for developing markers for phenolic compounds-based chapatti quality determination through biparental mapping population. The variation in the expression of the remaining nine genes in qRT-PCR and microarray data (Tables 5, 7) may be due to hypersensitivity of qRT-PCR technique as compared to microarray which sometimes differ the results of the two methods (Etienne et al., 2004). Ma et al. (2016) have studied the expression pattern of PAL, C4H, COMT, C3H, and 4CL which were higher in early stages of seed development and low in later stages of seed development. In our study also gene expression follows the similar trend except for few genes (Table 7). Both data revealed that the majority of genes showed down expression in the good chapatti variety in comparison to the poor chapatti variety. The gene expression level in the good chapatti variety was largely in agreement with that of phenolic compounds. The level of variation of 12 genes between good and poor chapatti quality wheat varieties is high and has potential in development of markers though biparental QTL or association mappings (Roy et al., 1999, 2010).

**Table 7:** **Differential expression (fold change) of 17 phenolic compound biosynthesis pathway genes at mid and late stages (14 DAA and 28 DAA, days after anthesis) of seed development between two diverse bread wheat varieties, “C306” and “Sonalika,” differing for chapatti (unleavened flat bread) quality**.

**Gene**	**Probe set**	**Fold change value (“C 306” vs. “Sonalika”)**	
		**14 DAA**	**28 DAA**
Phenylalanine ammonia lyase (PAL)	Ta.28046.1.A1_at	4.4, 1.1	4.0, 0.7
*4*-Coumarate CoA ligase (4CL)	Ta.5623.1.S1_x_at	−5.0, 1.6	4.1, 3.6
Ferulate 5 hydroxylase 1 (F5H1)	Ta.Affx.108591.1.S1_x_at	4.0, 2.0	−1.1, 0.1
Caffeoyl CoA O-methyltransferase (CCoMT1)	Ta.18653.1.S1_at	13.5, 2.9	−4.4, 0.7
Chalcone synthase (CHS)	Ta.9172.1.S1_at	−1.3, 0.0	2.3, 0.1
Cinnamoyl CoA reductase (CCR)	Ta.13990.1.S1_at	−87.3, 3.7	−12.9, 3.5
Cinnamate-4-hydroxylase 1 (C4H1)	Ta.9643.2.S1_x_at	−20.0, 5.2	−10.9, 2.6
Cinnamyl alcohol dehydrogenase (CAD)	Ta.13798.1.S1_at	−3.8, 0.1	−2.7, 0.2
1,2-rhamnosyltransferase (1,2 RhaT)	Ta.30441.1.A1_at	−3.1, 0.4	−4.2, 1.1
Benzoate-4-monoxygenate (B4MO)	Ta.254.1.S1_s_at	−5.3, 1.9	−62.6, 40.8
Caffeic acid -o-methyl transferase (COMT)	Ta.336.2.S1_at	−10.4, 2.9	130.1, 15.4
Flavone synthase (FNS)	Ta.9332.1.S1_x_at	−4.8, 2.0	−4.1, 1.3
Flavonol synthase 1 (FLS 1)	Ta.25327.1.A1_at	−3.8, 2.0	−38.8, 17.3
*p*-coumaroyl quinate/shikimate-3-hydroxylase (C3′H)	Ta.20769.1.S1_x_at	−6.9, 1.7	−8.4, 3.1
Quercetin- 3-O-glucoside l –rhamnosyltransferase (Q3-RhaT)	Ta.Affx.139860.1.A1_at	−8.1, 2.8	−6.1, 2.0
Vanillin dehydrogenase (VDH)	Ta.2955.2.A1_at	−33.2, 5.1	−10.4, 2.7
Vanillin synthase (VS)	Ta.Affx.107168.1.S1_x_at	−5.6, 1.4	−3.8, 0.4

*The data was produced on qRT-PCR*.

### Relationship between the comparative analyses of phenolic compounds and gene expression related studies

The relationship was drawn by considering the quantitative gene expression data of the late stage of seed development (i.e., 28 DAA) and quantitative measurement of phenolic compounds. The statistical significance of the relationship was not calculated as we used only two diverse bread wheat varieties for chapatti quality. However, it is found that the expression data of majority of the genes on qRT-PCR corroborated with that of PCs. The up-expression data of seven enzymes genes (7/17 = ~41%) such as B4MO, VDH, VS, C4H1, C3H, FLS, and Q3-RhaT in the poor chapatti quality variety was corroborated with the high content of their immediate products i.e., *4*-hydroxybenzoic acid, vanillic acid, vanillin, *p*-coumaric acid, caffeic acid, quercetin, and rutin, respectively (Table 7). In this study, we corroborated the expression data of only ~41% of genes with the content of their immediate products whereas Ma et al. (2016) found 100% of gene expression data with their immediate products in wheat. Similar relationship was drawn for the expression data retrieved from the microarrays of the late stage of seed development (i.e., 28 DAA) and quantitative measurement of phenolic compounds. It was found that the high expression of five enzyme genes such as B4MO, COMT, C4H1, FLS, and Q3-RhaT was corroborated with the high content of their immediate products such as *4*-hydroxy benzoic acid, ferulic acid, sinapic acid, *p*-coumaric acid, quercetin, and rutin, respectively in the poor chapatti quality. Therefore, in this study the expression level of only four enzyme genes (4/17 = ~24%), B4MO, C4H1, FLS, and Q3-RhaT, was corroborated on both qRT-PCR and microarrays as well as with that of their immediate products (PCs).

## Conclusion

An integrated approach of analytical tools such as UPLC-QTOF-MS and -MS/MS and functional genomics analysis such as quantitative gene expression analysis using wheat microarrays data and qRT-PCR tentatively identified about 80% (69/87) of plant phenolic compounds in bread wheat (*T. aestivum*) varieties, several of them were not reported earlier in wheat. This information can be useful for exploration of many plant PCs and their quantification in larger set of wheat germplasm and breeding population, which are otherwise limited in wheat. The expression profiling of forty-one phenylpropanoid pathway genes using microarray revealed temporal distribution pattern of their expression during seed development. This study can be extended in a large wheat germplasm and breeding population to understand genetics and molecular basis of PCs and their improvement using molecular breeding approaches such as QTL mapping and association mapping approaches.

## Author contributions

MS conducted in the experimental works and data analysis and manuscript writing. RS helped in the experimental work designing, data analysis, and manuscript write up. AS, PK, and AM helped in in experimental works and data analysis in genomics works. SS, SJ, and JS contributed in optimization of analytical experimental works on UPLC-MS/-MSMS. JR contributed in project designing, implementation, and manuscript write up.

### Conflict of interest statement

The authors declare that the research was conducted in the absence of any commercial or financial relationships that could be construed as a potential conflict of interest.

## References

[B1] Adom K. K. Sorrells M. E. Liu R. H. (2003). Phytochemical profiles and antioxidant activity of wheat varieties. J. Agric. Food Chem. 51, 7825. 10.1021/jf030404l 14664553

[B2] Bailly F. Cotelle P. (2005). Anti-HIV activities of natural antioxidant caffeic acid derivatives: toward an antiviral supplementationdiet. Rev. Curr. Med. Chem. 12, 1811–1818. 10.2174/0929867054367239 16029149

[B3] Bailly F. Queffelec C. Mbemba G. Mouscadet J. F. Cotelle P. (2005). Synthesis and HIV-1 integrase inhibitory activities of ccaffeicacid dimers derived from *Salvia officinalis*. Bioorg. Med. Chem. Lett. 15, 5053–5056. 10.1016/j.bmcl.2005.07.091 16183277

[B4] Bhatnagar T. Sachdev A. Johari R. P. (2002). Molecular characterization of glutenins in wheat varieties differing in chapatti quality characteristics. J. Plant Biochem. Biotechnol. 11, 33–36. 10.1007/BF03263131

[B5] Boerjan W. Ralph J. Baucher M. (2003). Lignin biosynthesis. Annu. Rev. Plant Physiol. 5, 519–546. 10.1146/annurev.arplant.54.031902.134938 14503002

[B6] Chen H. J. Inbaraj B. S. and Chen, B. H. (2012). Determination of phenolic acids and flavonoids in taraxacumformosanumkitam by liquid chromatography-tandem mass spectrometry coupled with a post-column derivatization technique. Int. J. Mol. Sci. 13, 260–285. 10.3390/ijms13010260 22312251 PMC3269685

[B7] Cheynier V. (2012). Phenolic compounds: from plants to foods. Phytochem. Rev. 11, 152–177. 10.1007/s11101-012-9242-8

[B8] Dajas F. (2012). Life or death: neuroprotective and anticancer effects of quercetin. J. Ethnopharmacol. 143, 383–396. 10.1016/j.jep.2012.07.005 22820241

[B9] Dao T. T. H. Linthorst H. J. M. Verpoorte R. (2011). Chalcone synthase and its functions in plant resistance. Phytochem. Rev. 10, 397–412. 10.1007/s11101-011-9211-7 21909286 PMC3148432

[B10] de Munter J. S. Hu F. B. Spiegelman D. Franz M. van Dam R. M. (2007). Whole grain bran, and germ intake and risk of type 2 diabetes: a prospective cohort study and systemic review. PLoS Med. 4:e261 10.1371/journal.pmed.0040261 17760498 PMC1952203

[B11] Dinelli G. Carretero A. S. Silvestro R. D. Marotti I. Fu S. Benedettelli S. B.. (2009). Determination of phenolic compounds in modern and old varieties of durum wheat using liquid chromatography coupled with time-of-flight mass spectrometry. J. Chromatogr. A 1216, 7229–7240. 10.1016/j.chroma.2009.08.041 19740468

[B12] Dinelli G. Segura-Carretero A. Di Silvestro R. Marotti I. Arraez-Roman D. Benedettelli S.. (2011). Profiles of phenolic compounds in modern and old common wheat varieties determined by liquid chromatography coupled with time-of-flight mass spectrometry. J. Chromatogr. A 1218, 7670–7681. 10.1016/j.chroma.2011.05.065 21683368

[B13] Etienne W. Meyer M. H. Peppers J. Meyer R. A. Jr. (2004). Comparison of mRNA gene expression by RT-PCR and DNA microarray. Biotechniques 36, 618–626. 15088380 10.2144/04364ST02

[B14] Evans A. M. DeHaven C. D. Barrett T. Mitchell M. Milgram E. (2009). Integrated, nontargeted ultrahigh performance liquid chromatography/electrospray ionization tandem mass spectrometry platform for the identification and relative quantification of the small-molecule complement of biological systems. Anal. Chem. 81, 6656–6567. 10.1021/ac901536h 19624122

[B15] Herrmann K. Weaver L. M. (1999). The shikimate pathway. Annu. Rev. Plant Physiol. Plant Mol. Biol. 50, 472–503. 10.1146/annurev.arplant.50.1.473 15012217

[B16] ICH (2005). Validation of analytical procedures: text and methodology, in International Conference on Harmonization (ICH) (Geneva), 17.

[B17] Imaida K. Hirose M. Yamaguchi S. Takahashi S. Ito N. (1990). Effects of naturally occurring antioxidants on combined 1,2-dimethylhydrazine and 1-methyl-nitrosourea- initiated carcinogenesis in F344 male rats. Cancer Lett. 55, 53–59. 10.1016/0304-3835(90)90065-6 2245410

[B18] Jan A. T. Kamli M. R. Murtaza I. Singh J. B. Ali A. Haq Q. M. R. (2010). Dietary flavonoid quercetin and associated health benefits an overview. Food Rev. Int. 26, 302–317. 10.1080/87559129.2010.484285

[B19] Jiang X. L. Liu Y. J. Li W. W. Zhao L. Meng F. Wang Y. S.. (2013). Tissue-specific, development-dependent phenolic compounds accumulation profile and gene expression pattern in tea plant [*Camellia sinensis*]. PLoS ONE 8:e62315. 10.1371/journal.pone.0062315 23646127 PMC3639974

[B20] Joseph D. Muralidhara K. M. (2013). Enhanced neuroprotective effect of fish oil in combination with quercetin against 3-nitropropionic acid induced oxidative stress in rat brain. Prog. Neuropsychopharmacol. Biol. Psychiatry 40, 83–92. 10.1016/j.pnpbp.2012.08.018 22960609

[B21] Livak K. J. Schmittgen T. D. (2001). Analysis of relative gene expression data using realtime quantitative PCR and the 2−ΔΔCT Method. Methods 25, 402–408. 10.1006/meth.2001.1262 11846609

[B22] Khanduja K. L. Avti P. K. Kumar S. Mittal N. Sohi K. K. Pathak C. M. (2006). Anti-apoptotic activity of caffeic acid, ellagic acid and ferulic acid in normal human peripheral blood mononuclear cells: a Bcl-2 independent mechanism. Biochim. Biophys. Acta 1760, 283–289. 10.1016/j.bbagen.2005.12.017 16459021

[B23] Kim K. H. Tsao R. Yang R. Cui S. W. (2006). Phenolic acid profiles and antioxidant activities of wheat bran extracts and the effect of hydrolysis conditions. Food Chem. 95, 466–473. 10.1016/j.foodchem.2005.01.032

[B24] Koshihara Y. Neichi T. Murota S. Lao A. Fujimoto Y. Tatsuno T. (1984). Caffeic acid is a selective inhibitor for leukotriene biosynthesis. Biochim. Biophys. Acta 792, 92–97. 10.1016/0005-2760(84)90287-X 6318834

[B25] Li L. Shewry P. R. Ward J. L. (2008). Phenolic acids in wheat varieties in the HEALTHGRAIN diversity screen. J. Agric. Food Chem. 56, 9732–9739. 10.1021/jf801069s 18921977

[B26] Ma D. Li Y. Zhang J. Wang C. Qin H. Ding H.. (2016). Accumulation of phenolic compounds and expression profiles of phenolic acid biosynthesis-related genes in developing grains of white, purple, and red wheat. Front. Plant Sci. 7:528. 10.3389/fpls.2016.00528 27148345 PMC4840273

[B27] McCallum J. A. Walker J. R. L. (1989). Proanthocyanidins in wheat bran. Cereal Chem. 67, 282–285.

[B28] Moore J. Liu J.-G. Zhou K. Yu L. (2006). Effects of genotype and environment on the antioxidant properties of hard winter wheat bran. J. Agric. Food Chem. 54, 5313–5322. 10.1021/jf060381l 16848511

[B29] Mpofu A. Sapirstein H. D. Beta T. (2006). Genotype and environmental variation in phenolic content, phenolic acid composition, and antioxidant activity of hard spring wheat. J. Agric. Food Chem. 54, 1265–1270. 10.1021/jf052683d 16478246

[B30] Nair R. B. Bastress K. L. Ruegger M. O. Denault J. W. Chapple C. (2004). The *Arabidopsis thaliana* REDUCED EPIDERMAL FLUORESCENCE1 gene encodes an aldehyde dehydrogenase involved in ferulic acid and sinapic acid biosynthesis. Plant Cell 16, 544–554. 10.1105/tpc.017509 14729911 PMC341923

[B31] Nicoletti I. Daniela M. D. De Rossi A. Taddei F. D'Egidio M. G. Corradini D. (2013). Identification and quantification of soluble free, soluble conjugated, and insoluble bound phenolic acids in durum wheat (*Triticumturgidum* L var durum) and derived products by RP-HPLC on a semimicro separation scale. J. Agric. Food Chem. 61, 11800–11807. 10.1021/jf403568c 24175612

[B32] Rao P. H. Leelavathi K. Shurpalekar S. R. (1986). Test baking of chapati– development of a method. Cereal Chem. 63, 297.

[B33] Roy J. K. Prasad M. Varshney R. K. Balyan H. S. Blake T. K. Dhaliwal H. S. (1999). Identification of a microsatellite on chromosome 6B and a STS on 7D of bread wheat showing association with preharvest sprouting tolerance. Theor. Appl. Genet. 99, 336–340. 10.1007/s001220051241

[B34] Roy J. K. Smith K. P. Muehlbauer G. J. Chao S. Close T. J. Steffenson B. J. (2010). Association mapping of spot blotch resistance in wild barley. Mol. Breed. 26, 243–256. 10.1007/s11032-010-9402-8 20694035 PMC2908432

[B35] Segura-Carretero A. Puertas-Mejıa M. A. Beltran R. Alonso-Villaverde C. Joven J. Dinelli G.. (2008). Selective extraction, separation, and identification of anthocyanins from *Hibiscus sabdariffa* L using solid phase extraction-capillary electrophoresis-mass spectrometry (time-of-flight /ion trap). Electrophoresis 29:2852. 10.1002/elps.200700819 18546170

[B36] Shewry P. R. (2009). Wheat. J. Exp. Bot. 60, 1537–1553. 10.1093/jxb/erp058 19386614

[B37] Singh A. Mantri S. Sharma M. Chaudhury A. Tuli R. Roy J. (2014). Genome-wide transcriptome study in wheat identified candidate genes related to processing quality, majority of them showing interaction (quality x development) and having temporal and spatial distributions. BMC Genomics 15:29. 10.1186/1471-2164-15-29 24433256 PMC3897974

[B38] Sivam A. S. Sun-Waterhouse D. Waterhouse G. I. Quek S. Perera C. O. (2011). Physicochemical properties of bread dough and finished bread with added pectin fiber and phenolic antioxidants. J. Food Sci. 76, 97–107. 10.1111/j.1750-3841.2011.02086.x 21535837

[B39] Theerasin S. Baker A. T. (2009). Analysis and identification of phenolic compounds in Dioscoreahispida Dennst. Asian J. Agric. Food Sci. 2, 547–560.

[B40] Vaher M. Matso K. Levandi T. Helmja K. Kaljurand M. (2010). Phenolic compounds and the antioxidant activity of the bran, flour and whole grain of different wheat varieties. Proc. Chem. 2, 76–82. 10.1016/j.proche.2009.12.013

[B41] Vanholme R. Demedts B. Morreel K. Ralph J. Boerjan W. (2010). Lignin biosynthesis and structure. Plant Physiol. 153, 895–905. 10.1104/pp.110.155119 20472751 PMC2899938

[B42] Verma A. R. Vijayakumar M. Mathela C. S. Rao C. V. (2009). *In vitro* and *in vivo* antioxidant properties of different fractions of Moringaoleifera leaves. Food Chem. Toxicol. 47, 2196–2201. 10.1016/j.fct.2009.06.005 19520138

[B43] Wang S. Liu L. Wang L. Hu Y. Zhang W. Liu R. (2012). Structural characterization and identification of major constituents in Jitai tablets by high-performance liquid chromatography/diode-array detection coupled with electrospray ionization tandem mass spectrometry. Molecules 17, 10470–10493. 10.3390/molecules170910470 22945027 PMC6268525

[B44] Zilic S. Arda S. Gul A. Marijana J. Vural G. (2012a). Distribution of phenolic compounds, yellow pigments and oxidative enzymes in wheat grains and their relation to antioxidant capacity of bran and debranned flour. J. Cereal Sci. 56, 652–658. 10.1016/j.jcs.2012.07.014

[B45] Zilic S. Serpen A. Akillioglu G. Gokmen V. Vancetovic J. (2012b). Phenolic compounds, carotenoids, anthocyanins and antioxidant capacity of colored maize (Zea mays L.) kernels. J. Agric. Food Chem. 60, 1224–1231. 10.1021/jf204367z 22248075

