# Supplementary material for: Comparative Analysis of Phenolic Compound Characterization and Their Biosynthesis Genes between Two Diverse Bread Wheat (Triticum aestivum) Varieties Differing for Chapatti (Unleavened Flat Bread) Quality
Source: Front Plant Sci. 2016 Dec 15;7:1870. doi: 10.3389/fpls.2016.01870 (PMC5156688; doi:10.3389/fpls.2016.01870)
Supplement: Supplementary file 1 [file DataSheet1.ZIP › Supplementary Table S1.doc]

**Supplementary Table ST1.** The molecular formula and masses of 87 phenolic compounds reported in plants were used for identification of the same in wheat using Peakview software (version 1.2.0.3).

| S.No. | Compound | Molecular Formula | Molecular mass (Daltons) | Classification |
| --- | --- | --- | --- | --- |
| 1 | Benzoic acid | C_7_H_6_O_2_ | 122.12 | Phenolic acid |
| 2 | 4-Hydroxybenzoic acid(*p*-Hydroxybenzoic acid) | C_7_H_6_O_3_ | 138.12 | HBA |
| 3 | 3,4-dihydroxy benzoic acid | C_7_H_6_O_4_ | 154.12 | HBA |
| 4 | 3,4,5-Trihydroxybenzoic acid (Gallic acid) | C_7_H_6_O_5_ | 170.12 | HBA |
| 5 | 4-Hydroxy-3-methoxybenzoic acid (Vanillic Acid) | C_8_H_8_O_4_ | 168.14 | HBA |
| 6 | Syringic Acid | C_9_H_10_O_5_ | 198.17 | HBA |
| 7 | 4-Hydroxy-3-methoxybenzaldehyde (Vanillin) | C_8_H_8_O_3_ | 152.14 | HBA |
| 8 | 1-(4-Hydroxy-3-methoxyphenyl)ethanone (Acetovanillone) | C_9_H_10_O_3_ | 166.17 | HBA |
| 9 | Gentisic acid | C_7_H_6_O_4_ | 154.12 | HBA |
| 10 | 2-Hydroxybenzoic acid (Salicylic acid) | C_7_H_6_O_3_ | 138.12 | HBA |
| 11 | 2,4-dihydroxybenzoic acid | C_7_H_6_O_4_ | 154.12 | HBA |
| 12 | 4-Hydroxybenzaldehyde (p-Hydroxybenzaldehyde) | C_7_H_6_O_2_ | 122.12 | HBA |
| 13 | Cinnamic acid | C_9_H_8_O_2_ | 148.15 | Phenolic acid |
| 14 | 4-Hydroxycinnamic acid (*p*-Coumaric Acid) | C_9_H_8_O_3_ | 164.15 | HCA |
| 15 | 3,4-Dihydroxy-trans-cinnamate (Caffeic Acid) | C_9_H_8_O_4_ | 180.15 | HCA |
| 16 | Ferulic Acid | C_10_H_10_O_4_ | 194.18 | HCA |
| 17 | 3-(4-Hydroxy-3,5-dimethoxyphenyl)prop-2-enoic acid (Sinapic acid) | C_11_H_12_O_5_ | 224.20 | HCA |
| 18 | *trans*-5-O-(4-Coumaroyl)-D-quinate (*p*-Coumaroylquinic acid) | C_16_H_18_O_8_ | 338.30 | HCA |
| 19 | 1,5-dicaffeoylquinic acid | C_25_H_24_O_12_ | 516.45 | HCA |
| 20 | *trans*-*p*-Coumaroyltartaric acid (Coutaric acid) | ‎C_13_H_12_O_8_ | 296.23 | HCA |
| 21 | Fertaric acid | C_14_H_14_O_9_ | 326.25 | HCA |
| 22 | 4-(3-Hydroxyprop-1-enyl)-2,6-dimethoxyphenol (Sinapyl alcohol) | C_11_H_14_O_4_ | 210.22 | HCA |
| 23 | 3-Caffeoylquinic acid (Chlorogenic acid) | C_16_H_18_O_9_ | 354.30 | HCA |
| 24 | Dihydroferulic acid isomer | C_20_H_18_O_8_ | 386.35 | HCA |
| 25 | Hinokinin | C_20_H_18_O_6_ | 354.35 | Lignan |
| 26 | Syringaresinol | C_22_H_26_O_8_ | 418.43 | Lignan |
| 27 | 2-(3,4-Dihydroxyphenyl)- 5,7-dihydroxy-4-chromenone (Luteolin) | C_15_H_10_O_6_ | 286.23 | Flavone |
| 28 | Apigetrin | C_21_H_20_O_10_ | 432.38 | Flavone |
| 29 | 4,5,7-trimethoxyflavone | C_18_H_16_O_5_ | 312.31 | Flavone |
| 30 | Apiin | C_26_H_28_O_14_ | 564.50 | Flavone |
| 31 | Sinensetin | C_20_H_20_O_7_ | 372.36 | Flavone |
| 32 | Apigenin | C_15_H_10_O_5_ | 270.23 | Flavone |
| 33 | Vicenin-2 isomer | C_27_H_30_O_15_ | 594.51 | Flavone |
| 34 | Apigenin-6-C-arabinoside-8-C-hexoside | C_26_H_28_O_14_ | 564.49 | Flavone |
| 35 | Apigenin-6/8-C-pentoside-8/6-C-hexoside | C_27_H_30_O_15_ | 594.51 | Flavone |
| 36 | Glycosylated and acetylated | C_25_H_26_O_13_ | 534.46 | Flavone |
|  | 3’,4’,5’-trihydroxy-3,7-dimethylflavone isomer |  |  |  |
| 37 | Lucenin-1/3 (luteolin-6/8-C-xyloside-8/6-C-glucoside) isomer | C_26_H_28_O_15_ | 580.50 | Flavone |
| 38 | 2-(3,4-Dimethoxyphenyl)-5,6,7,8-tetramethoxychromen-4-one (Nobiletin) | C_21_H_22_O_8_ | 402.39 | Flavone |
| 39 | Apigenin-6-C-beta-galactosyl-8-C-beta-glucosyl-O-glucuronopyranoside | C_33_H_38_O_21_ | 770.64 | Flavone |
| 40 | Methylisoorientin-2’-O-rhamnoside isomer | C_28_H_32_O_15_ | 608.54 | Flavone |
| 41 | Isovitexin-2’-O-rhamnoside | C_27_H_30_O_14_ | 578.52 | Flavone |
| 42 | Orientin/Isoorientin | C_21_H_20_O_11_ | 448.38 | Flavone |
| 43 | Glycosylated 3’,4’,5’-trihydroxy-3,7-dimethylflavone isomer | C_23_H_24_O_12_ | 492.42 | Flavone |
| 44 | 5,7-Dihydroxy-2-(4-hydroxy-3,5-dimethoxyphenyl)-4H-chromen-4-one (Tricin) | C_17_H_14_O_7_ | 330.28 | Flavone |
| 45 | Isovitexin | C_21_H_20_O_10_ | 432.37 | Flavone |
| 46 | Chrysoeriol | C_16_H_12_O_6_ | 300.26 | Flavone |
| 47 | Eriodictyol | C_15_H_12_O_6_ | 288.25 | Flavanone |
| 48 | Naringenin-7-O-glucoside | C_21_H_22_O_10_ | 434.39 | Flavanone |
| 49 | Hesperidin | C_28_H_34_O_15_ | 610.56 | Flavanone |
| 50 | Narirutin | C_27_H_32_O_14_ | 580.53 | Flavanone |
| 51 | 5,7-Dihydroxy-2-(4-hydroxyphenyl)chroman-4-one (Naringenin) | C_15_H_12_O_5_ | 272.25 | Flavanone |
| 52 | Naringin | C_27_H_32_O_14_ | 580.53 | Flavanone |
| 53 | 2-(3,4-Dihydroxyphenyl)-3,5,7-trihydroxy-4H-chromen-4-one (Quercetin) | C_15_H_10_O_7_ | 302.23 | Flavonol |
| 54 | Quercetin-4'-O-glucoside | C_21_H_20_O_12_ | 464.37 | Flavonol |
| 55 | Quercetin-3-O-glucuronide | C_21_H_18_O_13_ | 478.35 | Flavonol |
| 56 | 3,5,7-Trihydroxy-2-(4-methoxyphenyl)chromen-4-one (Kaempferide) | C_16_H_12_O_6_ | 300.26 | Flavonol |
| 57 | Kaempferol-3-O-glucoside | C_21_H_20_O_11_ | 448.36 | Flavonol |
| 58 | Myricetin-3 -glucoside | C_21_H_20_O_13_ | 480.37 | Flavonol |
| 59 | 3,5,7-Trihydroxy-2-(4-hydroxy-3-methoxyphenyl)chromen-4-one (Isorhamnetin) | C_16_H_12_O_7_ | 316.26 | Flavonol |
| 60 | Rutin | C_27_H_30_O_16_ | 610.51 | Flavonol |
| 61 | 3,5,7-Trihydroxy-2-(4-hydroxyphenyl)-4H-chromen-4-one (Kaempferol) | C_15_H_10_O_6_ | 286.23 | Flavonol |
| 62 | Catechin | C_15_H_14_O_6_ | 290.27 | Flavanol |
| 63 | Catechin gallate | C_22_H_18_O_10_ | 442.37 | Flavanol |
| 64 | Epigallocatechin gallate | C_22_H_18_O_10_ | 442.37 | Flavanol |
| 65 | Apiforol | C_15_H_14_O_5_ | 274.26 | Flavanol |
| 66 | Luteoforol | C_15_H_14_O_6_ | 290.26 | Flavanol |
| 67 | Taxifolin | C_15_H_12_O_7_ | 304.25 | Flavanonol |
| 68 | Taxifolin-7-glucoside | C_21_H_22_O_12_ | 466.38 | Flavanonol |
| 69 | 2-(3,4-Dihydroxyphenyl) chromenylium-3,5,7-triol (Cyanidin) | C_15_H_11_O_6_+ | 287.24 | Anthocyanidin |
| 70 | Cyanidin-3-galactoside | C_21_H_21_O_11_ | 449.38 | Anthocyanidin |
| 71 | Cyanidin-3- glucoside | C_21_H_21_O_11_ | 449.38 | Anthocyanidin |
| 72 | Cyanidin-3-rutinoside | C_27_H_31_O_15_ | 595.52 | Anthocyanidin |
| 73 | Peonidin-3-glucoside | C_22_H_23_O_11_Cl | 498.87 | Anthocyanidin |
| 74 | Pelargonidin-3-glucoside (callistephin) | C_21_H_21_O_10_Cl | 468.84 | Anthocyanidin |
| 75 | Apigenindin | C_15_H_11_O_4_+ | 255.24 | Anthocyanidin |
| 76 | Apigenindin – 5-O- glucoside | C_21_H_21_O_9_ | 417.38 | Anthocyanidin |
| 77 | 3-(4-Hydroxyphenyl)-1-(2,4,6-trihydroxyphenyl)propan-1-one (Phloretin) | C_15_H_14_O_5_ | 274.26 | Chalcone |
| 78 | Phloridzin | C_21_H_24_O_10_ | 436.41 | Chalcone |
| 79 | Astringin | C_20_H_22_O_9_ | 406.38 | Stilbenoid |
| 80 | Piceatannol | C_14_H_12_O_4_ | 244.24 | Stilbenoid |
| 81 | 3,5,4'-Trihydroxystilbene-3-O-β-D-glucopyranoside (*trans*-piceid) | C_20_H_22_O_8_ | 390.38 | Stilbenoid |
| 82 | Pinosylvin (double glycosylation) | C_26_H_32_O_12_ | 536.18 | Stilbenoid |
| 83 | Glycosylated pinosylvin | C_21_H_22_O_8_ | 402.39 | Stilbenoid |
| 84 | 6,7-Dihydroxy-chromen-2-one (Esculetin) | C_9_H_6_O_4_ | 178.14 | Coumarin |
| 85 | 2H-chromen-2-one (Coumarin) | C_9_H_6_O_2_ | 146.14 | Coumarin |
| 86 | Formononetin (Glycosylated and methylated) | C_23_H_24_O_9_ | 444.43 | Isoflavone |
| 87 | Procyanidin B-3 isomer | C_30_H_26_O_12_ | 578.52 | Condensed tannins |
