# Supplementary material for: Comparative Analysis of Phenolic Compound Characterization and Their Biosynthesis Genes between Two Diverse Bread Wheat (Triticum aestivum) Varieties Differing for Chapatti (Unleavened Flat Bread) Quality
Source: Front Plant Sci. 2016 Dec 15;7:1870. doi: 10.3389/fpls.2016.01870 (PMC5156688; doi:10.3389/fpls.2016.01870)
Supplement: Supplementary file 1 [file DataSheet1.ZIP › Supplementary Table S2.doc]

**Supplementary Table S2.** RT, MS, MS/MS data of 14 phenolic compounds identified and validated with their known standards. All the standards were run in negative ion mode except the 3 standards - hesperidin, salicylic acid and *4*-hydroxy benzoic acid in positive ion mode.

| **Phenolic compound** | **Mass** | **RT**  **(min)** | **Chromatogram showing RT** | **MS/MS** | **Precur-sor ion mass** | **MS/MS** |
| --- | --- | --- | --- | --- | --- | --- |
| Vanillic acid | 167.04 | 4.46 |  |  | 167 | 167, 152,123, 108 |
| Vanillin | 151.05 | 5.02 |  |  | 151 | 151, 136, 108, 92 |
| Ferulic acid | 193.06 | 5.57 |  |  | 193 | 193, 178,134 |
| *p*-Coumaric acid | 163.05 | 5.08 |  |  | 163 | 163, 119, 93 |
| Caffeic acid | 179.04 | 4.48 |  |  | 179 | 179, 135, 89 |
| Quercetin | 301.04 | 6.94 |  |  | 301 | 301, 273, 178, 151 |
| Rutin | 609.15 | 5.41 |  |  | 609 | 609, 301, 271 |
| Salicylic acid | 137.03 | 6.10 |  |  | 137 | 137, 93 |
| Chlorogenic acid | 353.09 | 5.18 |  |  | 353 | 353, 263, 190, 146, 102 |
| 2,4-dihydroxy benzoic acid | 153.03 | 3.60 |  |  | 153 | 153, 108 |
| Nobiletin | 401.13 | 6.56 |  |  | 401 | 401, 341, 189 |
| Hesperidin | 611.20 | 5.41 |  |  | 611 | 611, 465, 303 |
| Sinapic acid | 225.07 | 5.59 |  |  | 223 | 223, 208, 193, 149, 121 |
| *4*-hydroxy benzoic acid | 139.03 | 3.08 |  |  | 139 | 139, 121, 99, 75 |
