# Supplementary material for: Comparative Analysis of Phenolic Compound Characterization and Their Biosynthesis Genes between Two Diverse Bread Wheat (Triticum aestivum) Varieties Differing for Chapatti (Unleavened Flat Bread) Quality
Source: Front Plant Sci. 2016 Dec 15;7:1870. doi: 10.3389/fpls.2016.01870 (PMC5156688; doi:10.3389/fpls.2016.01870)
Supplement: Supplementary file 1 [file DataSheet1.ZIP › Supplementary Table S3.doc]

**Supplementary Table S3.** Normalized threshold cycle (ΔCт) of phenolic compound biosynthesis pathway genes during seed development. The data were taken at two stages of seed development (14 and 28 days after anthesis, DAA) in two bread wheat variety 'C 306' and 'Sonalika'. Wheat ADP Ribosylation Factor (ARF) was used as an internal control gene for normalization.

| **DAA** | **Gene** | **ΔCт** | |
| --- | --- | --- | --- |
|  |  | **‘C 306’** | **‘Sonalika’** |
| 14 | PAL 5 | 1.53 | 4.01 |
| 14 | PAL 5 | 2.47 | 4.21 |
| 14 | PAL 5 | 2.46 | 4.52 |
| 28 | PAL 5 | 0.77 | 3.05 |
| 28 | PAL 5 | 1.16 | 2.94 |
| 28 | PAL 5 | 1.00 | 2.93 |
|  |  |  |  |
| 14 | C4H1 | 10.83 | 6.66 |
| 14 | C4H1 | 11.37 | 6.67 |
| 14 | C4H1 | 10.77 | 6.76 |
| 28 | C4H1 | 6.30 | 3.20 |
| 28 | C4H1 | 6.39 | 3.02 |
| 28 | C4H1 | 6.73 | 2.95 |
|  |  |  |  |
| 14 | 4HCL1 | 5.38 | 3.30 |
| 14 | 4HCL1 | 6.60 | 3.83 |
| 14 | 4HCL1 | 5.69 | 3.68 |
| 28 | 4HCL1 | 1.98 | 2.80 |
| 28 | 4HCL1 | 0.79 | 3.84 |
| 28 | 4HCL1 | 1.79 | 2.99 |
|  |  |  |  |
| 14 | C3'H | 9.35 | 6.56 |
| 14 | C3'H | 9.22 | 6.86 |
| 14 | C3'H | 9.69 | 6.60 |
| 28 | C3'H | 8.10 | 5.19 |
| 28 | C3'H | 8.37 | 4.80 |
| 28 | C3'H | 7.60 | 5.04 |
|  |  |  |  |
| 14 | CCoMT1 | 4.82 | 8.63 |
| 14 | CCoMT1 | 5.66 | 9.05 |
| 14 | CCoMT1 | 5.55 | 9.56 |
| 28 | CCoMT1 | 4.57 | 2.53 |
| 28 | CCoMT1 | 5.23 | 3.29 |
| 28 | CCoMT1 | 5.56 | 3.18 |
|  |  |  |  |
| 14 | CCR | 12.76 | 6.26 |
| 14 | CCR | 12.49 | 6.03 |
| 14 | CCR | 12.60 | 6.22 |
| 28 | CCR | 6.71 | 3.14 |
| 28 | CCR | 6.74 | 3.42 |
| 28 | CCR | 7.26 | 3.19 |
|  |  |  |  |
| 14 | CAD | 5.13 | 3.20 |
| 14 | CAD | 5.24 | 3.33 |
| 14 | CAD | 5.20 | 3.24 |
| 28 | CAD | 4.39 | 2.95 |
| 28 | CAD | 4.40 | 3.08 |
| 28 | CAD | 4.48 | 2.98 |
|  |  |  |  |
| 14 | F5H1 | 0.81 | 2.74 |
| 14 | F5H1 | 1.28 | 2.32 |
| 14 | F5H1 | 0.91 | 3.51 |
| 28 | F5H1 | 2.08 | 1.81 |
| 28 | F5H1 | 1.86 | 1.58 |
| 28 | F5H1 | 1.77 | 1.77 |
|  |  |  |  |
| 14 | COMT | 6.91 | 3.18 |
| 14 | COMT | 6.70 | 3.79 |
| 14 | COMT | 7.09 | 3.72 |
| 28 | COMT | 1.03 | 7.88 |
| 28 | COMT | 0.82 | 7.83 |
| 28 | COMT | 0.93 | 8.12 |
|  |  |  |  |
| 14 | CHI | 12.25 | 8.36 |
| 14 | CHI | 11.28 | 7.36 |
| 14 | CHI | 12.58 | 8.55 |
| 28 | CHI | 6.73 | 2.81 |
| 28 | CHI | 6.12 | 2.19 |
| 28 | CHI | 6.55 | 2.50 |
|  |  |  |  |
| 14 | FLS1 | 10.05 | 8.90 |
| 14 | FLS1 | 10.54 | 9.07 |
| 14 | FLS1 | 10.85 | 8.16 |
| 28 | FLS1 | 9.80 | 4.59 |
| 28 | FLS1 | 10.19 | 4.36 |
| 28 | FLS1 | 9.57 | 5.08 |
|  |  |  |  |
| 14 | FNS | 12.97 | 11.02 |
| 14 | FNS | 13.44 | 11.62 |
| 14 | FNS | 14.21 | 11.39 |
| 28 | FNS | 12.37 | 10.18 |
| 28 | FNS | 12.12 | 9.78 |
| 28 | FNS | 11.11 | 9.69 |
|  |  |  |  |
| 14 | 1,2 RhaT | 8.47 | 6.78 |
| 14 | 1,2 RhaT | 8.20 | 6.45 |
| 14 | 1,2 RhaT | 8.11 | 6.70 |
| 28 | 1,2 RhaT | 6.01 | 3.95 |
| 28 | 1,2 RhaT | 6.31 | 3.93 |
| 28 | 1,2 RhaT | 5.63 | 4.00 |
|  |  |  |  |
| 14 | B4MO | 13.43 | 10.52 |
| 14 | B4MO | 13.40 | 11.27 |
| 14 | B4MO | 13.02 | 10.98 |
| 28 | B4MO | 12.47 | 6.95 |
| 28 | B4MO | 13.63 | 6.86 |
| 28 | B4MO | 12.28 | 7.24 |
|  |  |  |  |
| 14 | Q3-GRhaT | 9.65 | 6.21 |
| 14 | Q3-GRhaT | 9.47 | 7.05 |
| 14 | Q3-GRhaT | 9.76 | 6.77 |
| 28 | Q3-GRhaT | 6.79 | 4.00 |
| 28 | Q3-GRhaT | 6.29 | 3.40 |
| 28 | Q3-GRhaT | 5.78 | 3.85 |
|  |  |  |  |
| 14 | VDH | 13.64 | 8.72 |
| 14 | VDH | 13.60 | 8.68 |
| 14 | VDH | 13.84 | 8.55 |
| 28 | VDH | 10.26 | 7.00 |
| 28 | VDH | 10.08 | 6.34 |
| 28 | VDH | 9.71 | 6.67 |
|  |  |  |  |
| 14 | VS | 15.55 | 12.71 |
| 14 | VS | 15.17 | 12.98 |
| 14 | VS | 15.36 | 13.01 |
| 28 | VS | 13.38 | 11.65 |
| 28 | VS | 13.59 | 11.57 |
| 28 | VS | 13.77 | 11.75 |
