# Supplementary material for: Comparative Analysis of Phenolic Compound Characterization and Their Biosynthesis Genes between Two Diverse Bread Wheat (Triticum aestivum) Varieties Differing for Chapatti (Unleavened Flat Bread) Quality
Source: Front Plant Sci. 2016 Dec 15;7:1870. doi: 10.3389/fpls.2016.01870 (PMC5156688; doi:10.3389/fpls.2016.01870)
Supplement: Supplementary file 1 [file DataSheet1.ZIP › Supplementary Figure S1.doc]

**Supplementary Figure S1.** Total ion chromatograms (TIC) generated on UPLC-QTOF-MS showing peaks of phenolic compounds in the six extracts of the two diverse bread wheat varieties, ‘C 306’ (good) and ‘Sonalika’ (poor) differing for chapatti (unleavened flat bread) quality. Y-axis and X-axis represent **s**ignal intensity (counts per second, cps) and retention time (RT), respectively. The following six chromatograms in negative (ion) mode**:** (A) ‘C 306’ acidic, (B) ‘C 306’ alkaline, (C) ‘C 306’ free, (D) ‘Sonalika’ acidic, (E) ‘Sonalika’ alkaline and (F) ‘Sonalika’ free.
