# Supplementary material for: Comparative Analysis of Phenolic Compound Characterization and Their Biosynthesis Genes between Two Diverse Bread Wheat (Triticum aestivum) Varieties Differing for Chapatti (Unleavened Flat Bread) Quality
Source: Front Plant Sci. 2016 Dec 15;7:1870. doi: 10.3389/fpls.2016.01870 (PMC5156688; doi:10.3389/fpls.2016.01870)
Supplement: Supplementary file 1 [file DataSheet1.ZIP › Supplementary Material S1.doc]

**Supplementary Material S1.** The sequences of phenolic compound biosynthesis pathway genes highlighted for forward and reverse primers with green and pink color, respectively.

(1) >Phenylalanine ammonia-lyase (PAL 5); Ta.28046.1.A1_at; BQ161844

ACCCACTCATGCAGAAGATGCGTGCAGTTCTTGTGGAGAATGCCTTGGCAAATGGTGAGGCNGAGCGCGACGTGGAGACNTCNGTGTTTGCNAAGCTTGCCACGTTCGAGCNGGAGCTTCGGGCAGTGCTGCCAAAGGAGGTTGAGGCTGCCAGGAGCGCCGTGGAGAATGGCACTGCCNCACANCAAAACCGTATTGCCGAATGCCGGTCGTACCCNCTCTACCGATTCGTGCGCAAGGAGCTTGGAACGGAGTACTTGACCGGAGAGAAGACGCGGTCTCCTGGCGAAGAGGTGGACAAGGTGTTCNTTGCCATGAACCAGGGCAAGCACATCGATGCGCTNCTTGAGTGCCTCAAGGAGTGGAACGGCGAGCCCCTNCCTCTCTGCTAAATAGAGGATCGAGAAAGTGAAGAGTAGTGTGCTTCAGATTTCTGAAGGCTCTNATGATAATACTGTTTTTTCATTNTATATTCTAAAAGTTGATGTTTACAATGTTCTTCTAGAGCTGCCAATNTATTGCCAAAGATTGCAATTGCATGACTTGGTAGTGTTGGGTAGCCAGTAGAACTTTTATGATGTACGTAAGTTAAAANGGCANNNTGTGTNNNANTTTTCATGAT

(2) >Cinnamate 4-hydroxylase (C4H); Ta.9643.2.S1_x_at; CK211878

GATTCGGCCGAGGGGAGACGCTCCGGCTGCGCATGGCCATCCCGCTGCTGGTGCCCCACATGAACCTCCACGACGCCAAGCTCGCCGGCTACAACATCCCCNGCCGAGAGCAAGATCCTCGTCAACGCCTGGTTCCTCGCCAACAACCCGGAGCAGTGGAAGCGGCCCGACGAGTTCCGGCCGGAGCGCTTCCTGGAGGAGGAGAAGCACGTGGAGGCCAACGGCAACGACTTCAGGTACNTGCCCTTCGGCGTCGGCCGCCGGAGCTGCCCCGGCATCATCCTCGCGCTGCCCATCCTCGGCATCACCATCGGCCGCCTCGTCCAGAANTTCGTGCTCNNNGCNGCCGCCCGGNCAGGACAAGCTNGACACNACNGAGAAGGGNGGCCAGTTCAGCCTCCACATCCTCAAGCACTCCACCATCGTCGCCAAGCCCAGAGTGTTCTANAGCAGNNNNNNNNNNGACGTACGTGCTCTGGAGTTTGGACTTCCAGCATTTTGGNTTNGNTGNTGTNNNNCTTTCANGATTGTTCGCTCGTCCTAAGTATACTTGATTATAGTATCNNGTCACAGAGTAGACNCACGTTTCGTTAATGTTATCCGCAAGNAANCAGAGGAGCTGAAGAAATGTCGAACGGGGAGGAAAAGACTGGACGATTTCCNTCAGATGTACTCCTGATGCTGCCATAAATTAAATCCTCAAAATATTGATGATTTTTTCACAAANNAAANNANNNNNNANNNNNTTCTTGTAAATTTGTGGACTCATTTTGTAACGACCACTTGTGTGGAAAACTCGCCATGGGAAGCAACGCNTTTCGTNTTCCNNAAAAANNCNANANNANNNANNNNNNNNNAAAAAAAAAAAAAAGCCCACCTCGGCCGACCACGCTNNNNNNNNNNNNNNNNNNNNNNNNNNNNNNNNNNNNNNNNNNNNNNNNNNNNNNNNNNNNNNNNNNNNNNNNNNNNNNNNNNNNNNNNNNAGCT

(3) >4-coumarate coenzyme A ligase (4CL1); Ta.5623.1.S1_x_at; CK197632

GCGCCCATGGGCAANGNNCTNNNGNANNCNNNCNNGGCTANGATCCCNAACGCCGTGCTCNNCCNGNNNTNNGNNATGACGGAGGCCGGGCCGGTGCTGGCCATGTGCCTGGCGTTCGCCAAGGAGCCGTTCAAGGTCAAGTCCGGGTCGTGCGGCACGGTGGTNCNCAACGCGGGGCTCAAGATCGTCGACCCCGACACCGGCGCCTCCCTCGGCCGCAACCAGCCCGGCGAGATCTGCATCCGCGGCGAGCAGATCATGAAAGGTTACCTTAACGACCCAGAGTCCACCAAGAACACCATCGACAAGGATGGATGGCTGCACACCGGAGACATCGGCCTCGTCGACGACGACGACGAGATCTTCATCGTCGACAGGCTCAAGGAGATCATCAAGTACAAGGGCTTCCAGGTGGCGCCGGCGGAGCTCGAGGCCCTCCTCATCACGCACCCGGAGATCAAGGACGCCGCCGTNGTATCACTGAAGGACGATCTTGCTGGCGAAGTCCCGGTCGCCTTCGTNATGCGGATCGAAGGCTCCGAAATCACCGAGGATGACATCAAGAAATTCGTCGCAAAGGAGGTTGTTTTCTACAAGAGGATCCACAAGGTNTTCTTCACNGACTCCATNCCCNAAGAACCCTTCCGGCAAGATCCTGAGGAAGGANTTNGAGAGCCAGGCTCGCCGCCGGCATCCCNAGCTANGGGNTGCGNCCGTCGTCCGCTTCTCAGGCACTCAGCCAGCCAGCAGGGTGCTGTTATAGATCATGTAATATTGNTNGATCTCCANGAANTGGTCGGAGGAGGTTGAGTTTGTTGGTTCTTCCTGCNTGTGTAATGTTGATAAAAATGAAATGATATATGTAGATGCGTTGTTNTTGACNACAAGAACAAGGCTGGNTTCCAAAGGGTACNNNGNNNNNNNNNNNNCNNNNNNANNNTATCTTGTTNGNATCATANNANNNANNNNNNNNNNNANNANNNNNNNNNNNNNANNNNNNNNNNNNNNNNNNNNNNNNNNNNNNNNNNNNNNNNNNNNNNNNNNNNNNNNNNNNNNNNNNNNNNNNNNNNNNNNNNNNNNNNNNNNNNNNNNNNNNNNNNNNNNNNNNNNNNNNNNNNNNNNNNNCTAACTCGTATGGTAAGNTCACTANCGCACGGTCGGGTG

(4)>*p*-coumaroyl quinate/shikimate-3-hydroxylase (C3’H); Ta.20769.1.S1_x_at; CK194353

ANCCNNNNNNNNNNNNNNNNNCNNNNNNNNNNNNNNNNNNNNNNNNNNNNNNNNNNNNNNNNNNNNNNNNNNNNNNNNNNNNNNNNNNNNNNNNNNNNNNNNNNNNNNNNNNNNNNNNCCNNNNNNCNNNNCANNNCNNNAGCAGAGTCGCGGCAGNCACAATGGACGTGGCGTCGCTGCTCCCGTTCGCCATCGCGCTGGTGGCGATCCCGATCTCGCTGGCGCTGCTGGACCGGCTGCGGCTGGGNCGGCTGCCGCCGGGCCCGCGCCCGTGGCCGGTGNTGGGCAACCTGCGGCAGATCAAGCCGGTGCGGTGCCGCTGCTTCCAGGAGTGGGCGGNGCGGTACGGGCCCATCATCTCCGTCTGGTTCGGCTCCTCGCTCACCGTCGTCGTCTCCACCTCCGAGCTGGCCAAGGAGGTGCTCAAGGAGCACGACCAGCAGCTCGCCGACCGGCCCCGNAACCGCTCCACCCAGCGCTTCAGCCGCAACGGCCAGGACCTCATCTGGGCCGACTACGGCCCGCACTACATCAAGGTGCGCAAGCTCTGCAACCTCGAGCTCTTCACCCAGAAGCGCCTCGAGGCGCTGCGCCCCATCCGCGAGGACGAGGTCACCGCCATGGTCGAGTNCGTCCACCGNGCCGCCGCCGGCCCCGGTAATGAAGGAAAGCCATTGGTAGTGAGGAATCACCTTGCTATGGTGTCCTTCAACAATATAACAAGGCTAGCTTTTGGGAAGCGGTTCATGAATGCANATGGTGACATTGACGAAGAAGGGCAGGANTTCAAGNTTATTGTCAACAATGGNATCAAAATTGGTGCATCTCTNTCTGTTGCTGAATACATTTGGTACTTGAGATGGCTGTGTCCACTTAATGAGGAGCTTTACAANACCCACAATGAGAGNAGGGATCGTCTGACCAAGAAGATCATTGAAGAGCATGCACAGGCCCTCNNGGAGAGNGGTGCCAAACAGCACTTTGTGGATGCNCTGTTCACTCTCAGAGAGAAGTATGACCTTAGTGATGACACAGTTTTCGGACTTCTATGGGACATGATCACCGCCGGAATGGACACGACAGTCATATCAGTCGANTGGGCCATGGCAGAGCTGGTNNGGAACCCCAGGGTGCAGAAGAAGCTGCANGAGGAGCTGGACAGCGTGGTCGGCCGCGANCGCGTCATGTCCGAGACCGACTTCCNGAACCTGCCCTACCTGATGGCCGTCGTGAAGGAGTCCCTCCGCCTGCACCCGCCGACGCCGCTCATGCTGCCCCACAAGGCCAGCGCGAGCGTCAAGGTCGGCGGCTACAGCATCCCCAAGGGCGCCAACGTGATGGTGAACGTGTGGGCGGTGGCCCGGGACCCCAAGGTGTGGAGCAGCCCGCTGGAGTTCCGGCCGGAGCGGTTCCTGGAGGAGAGCATCGACATCAAGGGCAGCGACTTCAGGGTGCTGCCCTTCGGCGCNGGCCGGAGGGTGTGCCCCGGCGCGCAGCTCGGGATCAACCTCGTCGCCTCCATGATCGGNCACATGCTGCACCACTTCGAGTGGTCTCTGCCGGAGGGCGCCAGGCCGGAGGACATCAGCATGATGGAGTCCCCCGGGCTCGTCACCTTCATGGGCACGCCGCTGCAGGCCGTCGCCACGCCGCGCCTGGAGAATGAGGAGCTNTACAAGAGGGTCCCGGTNGAGATCTGAGCNNNNNNNNNTGCTNNNNTGNNNGNGNTGTTGCNNNNNNNGCTGCTTTCAGCTAGGTGCTGGATGGAGCTCGTGTTGTTGTAAATCGTGAGGTGTTTGACTGCCTTTTGAATGNATCTCATAAAGCGACGACGGCAGATCGCAGATCNTTTCTCCCTTTATTANNNGGTNTTCNTTCGNATATANTNNTTTTGTTTTTNTGTTAATAACAGTTTAACACAGCAAATCATTCTAGACAATGTATTTCTCCACATAATATANNNNNNNNNNNANTGAAAANNNNNNNNNNAAAAAAAAGNNNNNNNNNNNNNNNNNNNNNNNNNNNNNNNNNNNNNNNNNNNNNNNNNNNNNNNNNNNNNNNNNNNNNNNNNNNNNCTGCATCGTATGGTAANNCCACTAAGGCACGGGTTTGTTA

(5) >Caffeoyl-CoA O-methyltransferase 1 (CCoMT1); Ta.18653.1.S1_at; CK216386

CACCTGCTATTGCGNNNCNGCNNTNNGCNNNNNNNNNNNNNNNNNNNNNNNNNNTNNNNNNNNNNNNNNNNNNNNNNNNNNNNNNNNNNNNNNNNNNNNNNNNNNNNNNNNNNNNNNCNNNNNNNNNCCAACCCATNCCTCCTACTACCTCGGCACAACCAGCCCAAACAGAACTAGCGAGCAGAGCCATGGCGCCCAACGGAGACAACACCGTGGCCAACGTCCACAGCGGCATCGACAGCACCAACAAGACGCTGCTCAAGAGCGACGCCCTCTACACNTACATCCTCGACACCACCGTGTTCCCCCGCGAGCACGAGTGCATGCGCGACCTGCGCCTCATCACCGACAAGCACCCATGGGGNTACATGCAGTCGTCNTCGGACGAGGCGCAGCTGCTGGGGATGCTGATCAAGATGGCGGGCGCCAAGAAGACGATCGAGGTGGGCGTGTTCACGGGCTACTCGCTGCTGGCCACCGCGCTGGCGCTCCCGGAGGACGGCAAGGTGGTGGCCATCGACACCGACCGCGAGTGCTACGAGGTGGGNCNGCCCCTTCATCGAGAAGGCCGGCATGGCGCACAAGGTGGACTTCCGCGAGGGCACCGGCCTGGCGCGCCTGGACGAGCTNCNNNCGTCGAGGACGANGGCGCGGCNAGCTACGACTTCGCGTTCGTGGACGCGGACAAGCCCAACTACGTGCGCTACCACGAGCAGCTGCTGAAGCTGGTCCGCGTCGGCGGCACNATCATCTACGACAACACGCTNTGGGGCGGCACGGTGGCNCTGCCGGCGGGCACNCCCATGTCCGACCTCGACACCCGCTTCTCCGCCGCCCTCAGGGACCTCAACGCCAAGCTCGCCGCCGACCCGCGCATCGAGGTCTGCCAGCTCGCCATCGCCGACGGNGTCACCATCTGCCGCCGCATCGTCTAGNCGGGCGNNNNTCCGGCCCGGCCCGGCGTGATCGTGATCCGCCGNGCGCCTGCCGGCCTGCGTGCGTGCGTGGAGTCCATTTCCTTACCAGCCGCTTATCTTAATCAATTGTATTATTNTTTTTCCCTGCCAGCGCTGGCNNNNGCCGTGAAGCATCTGATGGTTTNAAANTTTCAATAATATTTTTGGCTTTTCAANNNNNANANNAGANGGAGGAGNGGCTGTACTTATGTACTGTAACTATACTANNNTATATGGTGTTCCGATGTCNNANNANNANNNNNNNNANNNNNNNNNNNNNNNNNNNNNNNNNNNNNNNNNNNNNNNNNNNNNNNNNNNNNNNNNNNNNNNNNNNNNNNNNNNNNNNNNNNNNNNTNNNNNNNNNNNNNNNNNNNNNNNNNNNNNNNGNNNGCNNNNNNNNNNNNNNNNNNNNNNNNNNNNNNNGGTTACCCTTAAAAAAAAAAAAAAAAAAAAAGNNNNNNNNNNNNNNNNNNNNNNNNNNNNNNNNNNNNNNNNNNNNNNNNNNNNNNNNNNNNNNNNNNNNNNNNNNNNNCTAAGTCGTANTGTAANNTANCTAACGCACCGTCNTGTG

(6) >Cinnamoyl-CoA reductase (CCR); Ta.13990.1.S1_at; CK212487

ATTCGGCANNNNNNNGCTGCCTAGCTCTACTTACTACCTGCGCAGTGTTGTGNGGTACTGTAGTAAGTAGCAGGGAAGAACCGTTCCGAGTACACAGCGTACTTTNGGACCTGTNGATACCTTGCTAGGCACCNGCTGCTCGCAACATCCCCGGATACGTCGTGNCAATCCGGAAGTAAGNCGAGAGGAAGAGAGAGATGCCGATCGAGGCGGCAGACAACGTNCCGGCCGAGCTTCCCGGCCACGGCCGGACGGTGTGCGTCACCGGCGCCGGCGGNTTCATCGCCTCGTGGCTCGTCAAGCGCCTCCTNCAGAAGGGNTACANCGTGCGNGGCACCGTTCGCAACCCNGTTGATCCGAAGAATGANCACCTNAGAGCCTTCGANNGGCGCCGCCGACCGCCTCNTCCTCCTCCGCGCCGACCTTATGGAGCCCGAAACCCTCGTCGAGGCGTTCACGGGCTGNGAGGGCATCTTCCACGCCGCTTCCCCNGTCACCGACGACCCTGAGAAGATGATCGAGCCGGCGATCCGNGGGACCAAGTANGTGATCACNGCAGCCGCGGACATGGGCATCAAGCGTGTNGTGTTCACGTCCACCATCGGCACGGTGTACATGAACCCCNACAGGGACNCCAGCAAGCCCGTCGACGACACCTGCTGGAGCGACCTTGAATACTGCANAAAGACAGCGAACTGGTACTGCTACGCGAAGACGGTGGCCGAGCAGGACGCGCTGGAGACGGCCNGGCNGCGCGGCATCGAGCTGATCGTGGTGAACCCGGTGCTGGTGCTGGGCCCGCTGCTGCANCCNACGGTGAACGCGAGCACGGAGCACGTGATGAAGTACCTGACGGGGTCGGCCAAGACGTACGTGAACGCGGCGCAGGCGTACGTGCACGTCAAGGANGTNGCCGAGGCGCACGTCCGCGTCTACGAGGCGCCCGGCGCGCACGGCCGCTACATCTGCGCCGAGGGCACCACGCTCCACCGCGGCGANCTCTGCCGCGTCCTCNGCAAGCTNTTCCCNGAGTACCCCGTACCCACCAAGTGCAAGGACGAGGTGAACCCACCGGTGAAAGGGTACAAATTCACNAACCAGAGGCTCAAGGANCTGGGNATGGAGTTCGTGCCGGTGCTGCAGAGNATATACGAGACGGTGAAGAGCCTCCAGGAGAAAGGGATGCTGCCGGTGCTACCGCCAGGCGACGACNTCCNAGACAACCTGCATGAGCAGCTCATGATGAAGCCCGCCCAGTTGCTACGTAATTAATTATACTCTACATTTGNTANNGATATNNGCTGTCGGCTAGTTAATTCTATGGGACATGNCTGTCCATTCGATCGGTCNTATATATGCNNNCNNNNNTACACGTATGCACTTTTAATTGGNGCATGCATGCGTGCGTGAGTGGGTGGGNNTTGGTTGGCGATCANATNNANNNATNGATCAGTGCTGTATGACGCACNNNANNNNCNCNNNAATGTTATATNCATNNANNGTGANATANATCCACGGACTACNATAAAAAAAANAAAAAAANNNCGGCCGCTCT

(7) >Cinnamyl alcohol dehydrogenase (CAD); Ta.13798.1.S1_at; CK161291

CAANNNCNNNNNCNNNNNNNNCNNNNNCNNNNNNNNNNNNNNNNNNNNNNNNNNNNNNNNNNNNNNNNNNNNNNNNNNNNNNNNNNNNNNNNNNNNNNNNNNNNNNNNNNNNNNNNNNNNNNNNNNNNNNNNNNCNNGNNNAAGNCANNTNNNNNNCCTNCCCAAATCCACNCANCNCNCNCCCGCGTCTCCTCCCGTTGCCGCANNAGCAAGAGAATGGGCAGCGTCGACGCCTCCGAGACGACGGTCACCGGGTGGGCCGCCAGGGACGCCACCGGCCACCTCTCCCCCTACANATACACNCTCAGGAAAACAGGNCCTGAAGANGTGGTGTTGAAGGTTAAGTACTGCGGCATCTGCCACACTGACNTCCACCAGGTCAAGAACGACCTCGGTGCTTCNAAGTACCCCATGGTCCCAGGGCATGAGGTGGTTGGCGAGGTGGTGGAGGTCGGGCCGGAGGTGAGCAAGTTCCGNGCCGGCGACGTGGTNGGNGTGGGNGTGATCGTGGGGTGCTGCCGCGACTGCCGGCCGTGCAAGGCCAACGTCGAGCAGTACTGCAACAAGAAGATCTGGTCGTACAACGACGTCTACACCGACGGCAAGCCGACGCAGGGCGGCTTCGCCTCCGCCATGGTCGTCGATCAGAAGTTCGTGGTGAAGATCCCNGCCGGGCTGGCGCCGGAGCAGGCGGCGCCGCTGCTGTGCGCGGGNGTGACGGTGTACAGCCCGCTGAAGCACTTCGGGCTCATGACCCCNGGCCTCCGCGGCGGCATCCTGGGCCTGGGCGGCGTGGGCCACATGGGCGTGAAGGTNGCCAAGTCCATGGGCCACCACGTGACGGTGATCAGCTCGTCCAACAAGAAGCGGGCCGAGGCCATGGACGACCTGGGCGCCGACGCGTACCTCGTCAGCTCCGACNCCGACCAGATGGCCGCCGCCGCCGACTCGCTGGACTACATCATCGACACNGTGCCCNGCNAAGCACCCGCTGGAGCCCTACCTGGCGCTGCTCAAGATGGACGGCAAGCTGGTGNTGATGGGCGTGATCGCGGAGCCGCTCAGCTTCGTGTCCCCCATGGTGATGCTGGGGAGGAAGACCATCACGGGNNAGCTTCATCGGGAGCATGGACGAGACGGAGGAGGTGCTCCAGTTCTGCGTCGACAANGGGCTCACCTCGCAGATCGAGGTCGTCAAGATGGACTACGTCAACCAGGCGTTCGAGAGGCTCGAGCGCAACGACGTNCGCTACCGCTTCGTCGTCGACGTCGGCGGGAGCAACATCGAGGACGCCGCCTGATCGANNNCGGCCGGNAAAANNAGAGNGNNNNNNNNCTAGACNGAGCGGCGAGTGCAATGACAGTATCATGTGTGCCCGTGNCATTTGCCTGNACTTGATGCAGTACATGTTNGTGACCTGTTTCCGTTTTCNATCAGAGTNGCGCGCGTTCGTTTCGGTGGAGATGCATGTTTTGCTGTTAATCCGGCCTTAANGACTATGATATATGTNNATGGAGTTATNTCTACTCTTAATNNCTGAGTTGGTTGAATAGTACGNTTTANNNNNANNNNANNANNNNNNNNNNNNNNNNNNNNNNNANAANAANNNNNNNNNNNNNNNNNNNNNNNNNNNNNNNNNNNNNNNNNNNNNNNNNNNNNNNNNNNNNNNNNNNNNNNNNNNNNNNNNCNNANNNNNNNNNNANGCNNCNNAACNANNGNNGNNTNNA

(8) >Ferulate 5-hydroxylase 1(F5H1); TaAffx.108591.1.S1_x_at; CD877874

CTCGTGCCGAANNNNGCNNNNNNNNCGTNNNNNNNNATCAACCTAAAACCCACACCGTCGAATCGAACACCCAAGAAAAGCCAAAAACAANTTAGAGATTCTAGTTCGAGTATCNNTCAATGGNGGNTTTGGCCAAGACCGCCATGGAATGGCTCCAAGATCCACTGAGCTGGCTGTTCGTCGCCTCGGTGGTCTTCGTGGTGCTGCNGCGGCGCCNGCGNGGCAAGNCGCCGCCGTTTCCTCCNNGGGCCGAATCAGCTGCCGNTCGTCGGCAACATGTCGATGATGGACCANGCTGACCCACCGGGGCCTCGCGGCGCTGGCGAAGCAGTACGGCGGCCTTCTCCANCTCCGCCTCGGNAAGCTCCATGCCTTCGCCNGTGTNCGACGCCGGAGTACGCCCGGGANGGTGCTGCANGGCGCANGACGGNGCCTTCTCCAACCGGNCCGGNNANCATCGCCATCGCCTANCTCANCTACNANCGNTGCCGACATGGNGTTCGCGCANTANGGGNCCTTNTGGNGNNANATNCNCAANGCTGTGCGTGATGAANCTCTTCAGCCGGCGCCGTCCGGAGACGNGGGTCGNNGNGCGCGACGAATCCGCGGCGCTCG

(9) >Caffeic acid –O-methyl transferase (COMT); Ta.336.2.S1_at; BJ252644

CAAACATGGGCTCCACCGCAGCCGACATGGCCGCCTCCGCCGACGAGGAGGCGTGCATGTATGCTCTCCAGCTCGTCTCGTCGTCGATCCTCCCGATGACGCTCAAGAACGCCATCGAGCTGGGTCTCCTGGAGACCCTGGTGGCCGCCGGCGGCAAGCTGCTGACGCCCGCCGAGGTGGCAGCCAAGCTCCCGTCCACGGCGAATCCCGCCGCGGCGGACATGGTGGACCGCATGCTCCGGCTGCTGGCCTCGTACAACGTGGTGTCGTGCACGATGGAGGAGGGCAAGGACGGCCGGCTGTCCCGGCGGTACGGCGCCGCGCCCGTGTGCAAGTTCCTCACCCCCAACGAAGACGGCGTCTCCATGGCGGCGCTCGCGCTCATGAACCAGGACAAGGTCCTCATGGAGAGCTGGTACTACCTGAAGGACGCGGTCCTTGACGGCGGCATCCCGTTCAACAAGGCGTACGGGATGTCGGCGTTCGAGTACCACGGCACGGACCCGCGCTTCAACCGCGTCTTCAACGAGGGGATGAAGAACCACTCCATCATCATCACCAAGAAGCTCCTCGAGGTCTACAANGGCTTCGAGGGCNTCNGNANCATCGTCGACNTNGGCNGNGGNNNNNTGGGCGCCATCGTCGCCGCCTACCCGGCCATCAAGGGCATCAACTTCGACCTCCCCCACGTCATCTCCGAGGCGCCACCGTTCCCGGGCGTCACCCACGTCGGCGGCGACATGTTCCAGAAGGTGCCCTCGGGCGACGCCATCCTCATGAAGTGGATCCTCCACGACTGGAGCGACGAGCACTGCGCGACGCTGCTCAAGAACTGCTACGACGCGCTGCCGGCGCACGGCAAGGTGGTGCTCGTGGAGTGCATCCTGCCGGTGAACCCGGAGGCCACGCCCAAGGCGCAGGGGGTATTCCACGTCGACATGATCATGCTCGCGCACAACCCCGGCGGCAGGGAGAGGTACGAGAGGGAGTTCGAGGCCCTGGCCAAGGGCGCCGGCTTCAAAGCCATCAAGACCACCTACATCTACGCCAACGCATTTGCCATCGAGTTCACCAAGTAGATCCATGCCAACCGTCCACCGCTCGCTTTGAGACCATCTTCTTCTTCCTCGCTGGCGCTGCTGAATATGTACTTTGGCTTGCTGTTTCCTCTGTTTTCCTAATTTTGTCATCCTAGCTCTGAATCATTCTGAATGCTACTGGTTGTGCCGTCGATCTACCCATTCGAAATGTACTGTACTATTAATAGTGCTGCTCTTAAAGNNNNNNNGTGATGCAATGANNNANNNNNNNNNNNNNTTNATTATAGGAAGAGT

(10) >Chalcone synthase (CHS); Ta.9172.1.S1_at; CA618801

CCACGCGTTCCGAATNAGTGGGCCATNCGACAAAGAAAGTACCAGCAAGTACTCGGAAACATGATCGAATCCTTGAGTTGACTTGAGAGCTAATCTCTACATTAGTTTCTTCAAATAGAAATTAACCTCCTACATTAAAGAGAGAGAGAAACCCAAATGTAGACATCGCTTGCACACCTAATTAACCCACGAATACTTGTCTCCTGTCTGGTTTGCCTTTTCTTGGTCTGGACACGCGAGCGAGCTAACNNNNNNNNNCNNNGNGNNNTNNNNNNNNNTGNNGNNGACAAGTCGCAGATNNNGNAAGAGNTANATGCACCTNACGGAGGAGATCCTNCAGGANAACCCCAACATGTGCNCCTACATGGCGCCNTCCCTGGACGCGCGCCAGGACATCGTCGTCGTNGAGGTCCCCAAGCTCGNGNAAGGCGGCCNGCGCAGAAGGCCATCAAGGAGTGGGGCCAGCCGCGCTCCAAGATCACCCACCTCGTCTTCTGCACCACCTCCGGCGTCGACATGCCCGGCGCCGACTACCAGCTCACCAAGATGCTCGGCCTCCGCCCCTNCGTCAAGCGCCTCATGATGTACCAGCAGGGCTGCTTCGCCGGCGGCACCGTGCTCCGCCTCGCCAAGGACCTCGCCGAGAACAACCGCGGCGCGCGCGTGCTCGTCGTCTGCTCCGAGATCACCGNNGTCACCTTCCGCGGCCCGCACGAGTNCCACCTCGACTCNNTNGTCGNCCAGGCGCTCTTNGGCGATGGNGCGGCCGCGGTGATCNTCGGCGCCGACCCCGACGTGTCCGTGGAGCGTCCCCTGTTCCAGCTCGTGTCCGCGAGCCAGACNATCCTGCCGGACTCNGAGGNCGCCATCGACGGCCACCTCCGGGAGGTCGGNCTCACCTTCCACCTGCTCAAGGACGTGCCCGGGCTCATCTCCAAGAACATCGAGCGNGCNCTAGAGGAGGCCTTCAAGCCACTNGGCATCGACGACTGGAACTCCGTCTTCTGGATAGCGCACCCAGGCGGCCCNGCNATCCTTGACATGGTNGAGGCCAAGGTAAACCTCNACAAGGAGCGGATGCGCGCNACCAGGCATGTCCTCTCCGAGTACGGCAACATGTCCAGNGCCTGTGTCCTCTTCATCATGGATGAGATGCGCAAGCGCTCNGCCGAGGATGGCCACGCCACNACCGGCGAGGGAATGGACTGGGGCGTCCTCTTCGGCTTCGGCCCCGGCCTCACGGTGGAGACCGTCGTCCTCCACAGCGTCCCCATCACNGCCGGNGCNACCGCNTGATCNNNGCTCCTTCTCCATTTATNNTCNNCNTNNCATGGAAAANNNNNACNNANNACNTACAGCNNNTTCTACNNTGCCGANNNNNGTTTGTACNATATATATTCTACCGTNNNNGCGTGGTTTGTANNNTGTGTTGGTAGANNNTGCATTCNNNNTNCTGTTAGTCTGTGTCGCGCACGCCGTNGCATACTTACTAGTAGTAACACAACCGAGCTTTTACAGGTTTATTATAAGCAAAGTGTGGTCATAATGTGGAGTGTGAGGTGTATTACCNTGTGTACGAAAAGATNGGCTGTTCAATTTGANCCTTATGTGGTTGGAAANCAAANNNNNANNNNNTNNNANNTNNNNTNANNNANNTATNCNNCNCNANNNNNNNNNNTAAACTCTAC

(11) >Flavonol synthase1 (FLS1); Ta.25327.1.A1_at; CD454732

CGATTCGGCACGAGCTGACGCAAGCCGGCGCGAGAGGAGATCGAGGTGCGTAATACTCCGCCGGCTCGATCGGGGGAGGCGACGGNNANGNNNNNGACGGCGGCGCANAGCGTGCAGGCGCTGGCGTCCTCGCTGGGCGCGCTCCCGCCCGAGTTCGTGCGGCCCGAGCACGAGCAGCCGCGCGCCACCACGTTCCGCGGGGCCGCGCCGCCGGAAATCCCCGTCATCAACATGTCCTCGCCGGGCGCCTGGCGCCGCATGGCGGAGGCCGCGGCGGAGTGGGGCATCTTCCAGGTGGTGTGCCACGGGGTGCCGGCTGAGGCCGTGGCGGAGCTGCAGCGCGTGGGCCGCGGGTTCTTCGCGCTGCCGCAGGAGGAGAAGCAGCGGTACGCCATGGACCCGGCCGAGGGGAAGACCGAGGGCTACGGCTCCACGCTGCGGAAGGGCGACCTCGAGGGCAAGAAGGCCTGGGCCGACTTCCTCTTCCACAACGTCGCGCCGCCGGCCGCCGTGAACCACGCCGTCTGGCCGGAGAGCCCCGAGGGGTACAGGGAGGNCAACGAGGCCTACTGCGGCCACATGCTGCGGCTCACGCGGGAGCTGTTCGGGCGCCTCTCGGCGGAGCTGGGCCTGCAGGANGGCGCCATGGCGGAGGCGTTCGGCGGCGACGACGTGGTGCTCCTCCAGAAGATCAACTTCTACCCGCCGTGCCCNCAGCCGGAGCTCGCGCTCGGCCTCGCGCCGCACACCGACATGAGCACGCTCACCGTCCTCCTGCCCGACGAGGTGCAGGGCCTCCAGGTGTTCAAGGACGGATGCTGGTACGACGTCAACTACGTGCCCGGCGCCCTCATCATCCACATCGGCGACCAGATCGAGATCATGAGCAACGGGAGGTACAAGGCCGTGCTGCACCGGACGACGGTGAGCCGGGAGAAGACGCGGATGTCGTGGCCGGTGTTCGTGGAGCCGCCGCCGGAGCACGCCGTCGGGCCGCACCCGCAGCTCGTCACCGACGAGTTCNCGGCCAAGTACAAGGCCAAGAAGTTCAAGGACTACAAGTACTGCAAGATCAACAAGCTACCACAGTAGCCAAATCCTTCAGACAGAGACCTTTAACCAATAATCCCCTTGTTCATATGTACTGTAATCCTACATTTCATGCGTGTCANTTTGGNTTTNNTNTNNNAAAANNNNNNNNNNNNNNNNNNN

(12) >Flavone synthase (FNS); Ta.9332.1.S1_x_at; BQ789066

CCACGCGTNCGNNNNNNNGCCAGCTCAAGCTTGAAGACTTCACTTAGCCACAGNNACNTGCATCNANCNTNANCNNACCNGNCCGGNNNNNCNGCNTCGATCCNAATGGAGCTTCCTCAGCTNGCGTCCTTCCTCGGCGTGGTGCTCGCCACGGTGCTCTTCNTCAAGGCTGTCCTNNGCNGCCGCCGCCNNNAGTACAACCTCCCGCCGGGCCCCAAGCCNTGGCCGATCATCGGCAACCTCAACCTCATCGGCACGCTCCCGCACCGCTCCATCCACGCGCTNTCCAAGNAAGTACGGCCCGCTCATGCAACTCCAGTTCGGCTCCTTCCCNGTCGTCGTCGGCTCCTCCGTGGAGATGGCCAAGTTCTTCCTCAAGACCCACGACGTGGTGTTCACCGANCGCCCCAAGACCGCCGCCGGCAGGTACACCACCTACAACTACAGCGACATCACCTGGTCCCCCTACGGCGCCTACTGGCGCCAGGCCCGCAAGATGTGCCTCACCGAGCTCTTCAGCGCCAAGCGNCTCGAGTCGTACGAGTACATCCGCAGGGAGGAGGTNCTCGCCCTNCTCGGCGACCTGTANTCGNGGNCGGCGCCGGNGCCGGCCGCGTGGTGGTGCTCAAGGACTACCTGTCCACGGTGAGCCTGAACGTGATCACGCGCATGGTGATGGGCAAGAAGTACCTGGAGAAGGAGGTGANGGACGAGGCAGGGGCGGTGATCACGACGCCCGAGGAGTTCAAGTGGATGATCGACGAGCTGTTCCTGCTCAACGGCGTGCTCAACATCGGCGACTCCATCCCGTGGCTGGACTGGATGGACCTGCAGGGGTACATCAAGAGGATGAAGAAGCTGAGCAAGATGTTCGANCGNNNNNNNGNNCNNGNNGNNGACGAGCACAGCGAGNGGCGTNGGCGCGAGGGGGAGAGCTTCGNTGGTGAAGGACATGGTGGACGTGCTGCTGCAGTTCGCCAGCGATCCCGGTCTNGAGGTCAAGCTCAACAGAGAGGGCGTCAAGGCTTTCACTCAGGACCTCATTGCNGGCGGNACNGAGAGCTCCGCGGTGACNGTGGAATGGGCCCTCTCAGAGCTCCTGAAGAAGCCAGAGGTGCTTGCCAAAGCCACNGAGGAGTTGGACCGNGTGGTGGGGCGAGGNCCGATGGGTCACCGAAAAGGACATGNNGAACCTTCCNTATGTGGATGCCATNGTCAAAGANANCNATGCGNTTGCANCCCGGTAGCGCCNGATGCTTGTACCCCNNGCCTTTCNCGNGAGGACACGTCCATCAANGGCTANGACATNCCNGCCGGCACACGGGNTGCTAGTCATGGTNTGGTCTATTNGGNCGCGACCCAGAGTTNTGGNAAGCGCCGGAGGAGTTCATGCCNGANCGNTTCCTCGGNNAGCANGCTCGANGTCAAGGGGCAGGATTATGAGNTGCTTCCATTCGGGTCGGGACGNAGGATGTGCCCCNGGGTATAGTCTTGGGTTGAAGGTGATCCAGGTAAGNCTNGCGAACCTCCTGCACGGGTTCACGTGGAGGCTCCCTGATGGCGTGGAGCTGAGCATGGAGGAGATCTTCGGACTGTCNACGCCACGCAAGTNCCCGCTGGAGGCNGTCNTGGAGCCCAAGCTCCCNGCTCACCTCTACGNNGAGGCTTGAATATNTGATACATGTGTGCNCTCNTGTGTTGAGTAATTATTACTNCTTGTGTTGTTNTATTTTTNCTNTANTNTTTTTCTAGTATTNTATNNTNNNNNNTGTNNNNNNNNNNNTNNNGANNNNNNANGANGGGTGTAAAAAAATGTTTGGTAATTGCTAATTCAATAAGTGAAATAATGGGATTTNNTCNNNNNAAAAAAAAAAAAAANNNNANANANNNNNNNNNNNANNANNNNAATCA

(13) >1,2 Rhamnosyl transferase (1,2 RhaT); Ta.30441.1.A1_at; CK205858

TGGAGTCCACCAACCGGCCGTTCTTCGTCGTGCTCAACTTCCCCAAGGGGCACGGACACCGACGCGGAGCTGGCGAGGTGCACNCCGCCGGGGTTCGCCGGGAGGACCAAGGGCAGGGGCGTGGTGCACACGGGGTGGGTGCAGCAGCAGCACATCCTGCGGCACCGCAGCGTGGGCTGCTTCGTGAACCACGCCGGGCTGAGCTCCGTGGTGGAGGGACTCGTCGCCGGCTGCCGGCTGGTGCTGCTGCCGATGAAGGGCGATCAGTACCTCAACGCGGCGCTGTTCGCGCGTGACCTTCGCGTCGGGGCGGAGGTGGCGCGGCGCGACGGCGACGGGTGGTTCGGGCGCGCGGACNTGANCGACNCCGTGGACACGGCGATGGCGGACGGGTGGGAGGGCCGAGGAATTAAATGGAGGGAGTTCTTGACGGACGACGCCGTGCAGAAGAGGTTGGCGGNCGATTTCGTCAGGGATTTCAAGAACTTCGTGAGGGCCTGAAAAGATGATGTACGCTTTGCTTGCTACTCCTACTAGGTCCCGTGTCCATGTCCATGGAGTCNAATNAAATGTGAACGATTNTG

(14) >Benzoate 4-monoxygenase (B4MO); Ta.254.1.S1_s_at; AF123609.1

CCACGCGTCCGTGTGGAGTCACTTATTACGCCCAGCCCAGCCCAGCCAGCACCTTAACTCGCCGATCCACCCCTTGGATCCACTCTACCCAGCTCGCTAGCCAGCGGGGTANATACACGCACGCACGTACGCGCGTACGTACACTNGCAGAGCTTGCTTCAGGGAGGNNNNCCGGCAATGGAGGTGGGGACGTGGGCGGTGGTGGNGTCGGCGGTGGCCGCGTACATGGCGTGGTTCTGGCGGATGTCNCGCGGGCTGCGCGGGCCGCGGGTNTGGCCCGTGCTCGGCAGCCTGCCNGGCCTGGTGCAGCACGCCGAGGACATGCACGAGTGGATCGCCGGCAACCTGCGCCGCGCGGGCGGCACNTACCAGACCTGCATCTTCGCNGTGCCCGGGGTGGCGCGCCGCGGCGGNCTGGTCACCGTCACCNNCGNNNCNNGCAACCTGGAGCACGTCCTNAAGGCGCGCTTCGACAACTACCCCAAGGGCCCCTTCTGGCACGGCGTCTTCCGGGACCTGCTCGGCGACGGCATCTTCAANTCCGACGGCGACACCTGGCTCGCGCAGCGCAAGACGGCCGCGCTCGAGTTCACCACCCGCACGCTCCGGACNGCCATGTCCCGCTGGGTCTCGCGCTCCATCCACGGCCGCCTCCTGCCCATCCTGGCCGACGCGGCCAAGGGCAAGGCGCANGTGGATCTCCAGGACCTCCTCCTCCGCCTCACCTTCGACAACATCTGCGGCCTGGCCTTCGGCAAGGACCCGGAGACGCTCGCCCAGGGCCTGCCGGAGAACGAGTTCGCCTCCGCGTTCGACCGCGCCACCGAGGCCACGCTCAACCGCTTCATCTTCCCGGAGTTCCTGTGGCGCTGCAAAAAGTGGCTGGGCCTCGGCATGGAGACCACGCTGACCAGCAGCATGGCCCACGTCGACCAGTACCTCGCCGCCGTCATCAAGAAGCGCAAGCTCGAGCTCGCCGCCGGCNACGGCNAATGCGACACGGCGGCGACGCACGACGACCTGCTCTCCCGGTTCATGCGGAAGGGTTCCTACTCGGACGAGTCGCTCCAGCACGTGGCGCTCAACTTCATCCTCGCCGGCCGCGACACCTCCTCCGTGGCGCTCTCCTGGTTCTTCTGGCTCGTGTCCACCCACCCTGCGGTGGAGCGCAAGATCGTGCGCGAGCTCTGCTCCGTTCTCGCCGCGTCACGGGGCGCCCATGACCCGGCATTGTGGCTGGCGGAGCCCTTCNCNTNCNANGNGNNCNNNNGCNNNNNCTACCTCAAGGCGGCGCTGTCGGAGACCCTCCGCCTCNANCNNNCNNNCNNNNAGGNCTCCAAGCACGTCGTCGCGGACGACTACCTCCCCGACGGCACNTTCGTGCCGGCCGGGTCGTCGGTCACCTACTCCATATACTCGGCGGGGCGCATGAAGGNGGTGTGGGGGGAGGACTGCCTCGAGTTCCGGCCGGAGCGATGGCTGTCGGCCGACGGCACCAAGTTCGAGCAGCANGACTCGTACAAGTTCGTGGCGTTCAACGCCGGGCCGAGGGTGTGCCTGGGCAAGGACCTAGCCTACCTGCAGATGAAGAACATCGCCGGGAGCGTGCTGCTCCGGCACCGCCTGACCGTGGCGCCGGGCCACCGCGTGGAGCAGAAGATGTCGCTCACGCTCTTCATGAAGGNCGGGCTACGGATGGAGGTACGTCCGCGCGACCTCGCCCCCGTCCTCGACGAGCCCTGCGGCCTGGACGCCGGCGCCGCCNNNACCGCCGCCGCAGCNAGTGCCACAGCGCCNTGCGCGTAGAAGACCTGGCACNNNNNCGGNCACGCGCCATGCATGATTCGTGNGTGCTAGCTGTTGAAGGGACGCCGGACATTGAATGTGTAGATAGGGCAGCAGTGCAAGACCGTAAGTAAAATTGATGATGGGTTTGGTGACAACATTNAAGNCACTCCTTTCCAGAATTTACGACCCGGATAGGAGAAACAGGGAAACTTTGCAGATCACAACACAAGATCTAGCCAGCCGGGGATCTGATCTGATNTGNGTCTGCTCNGAGCACGGGTGCATGGGAGACCAAGGAGGAAANCANAAAATAACAGAAACNGAGTGAGCAATATTTGTGATTGTAGCCACGGGANAGAGAGAGGAGTAATTAGTAATTCAGATTTGTTTGCAGTAGCTCGGTGTTGGTGACCAGATCATAGCCAACTAGGCTATTCTATTCTATTCTATTNTNNNNNNNGANNTNNNNNNNNAAANNNNNNNANNNNNANNCTATTCTTAGAGAGTTCATAT

(15)>Quercetin-3-O-glucoside L–Rhamnosyl transferase (Q3-GRhaT);

TaAffx.139860.1.A1_at; BG904338

GACAGGACGAGGGCACGCGGGATGGTGGTTTCTTCCTGGGCGCCGCAAGTGGAGGTGCTCCGGCACACGGCGACCGGCGCGTTCGTGACGCGCTGCGGATGGAACTCGACGCTTGAGGCTGTTGTCGCCGGGGTGACGATGCTGAGCTGGACCATGTACTCGGAGCAGGGGATGAACAAGGTGTTGGTGGTGGAGCACATGAAGCTTGAGGTGGCCATGGACGGTTCCCACGAGTGGACCGTGAAATCTAGGAAGGTAGAGGGGAAAGTGAGCCTGGTCATGGAGTCCGAGCATGGGAATCAGTTCAGGTAGAGGATGGCGATAGCGAAAGTGATTGCAGCCGATGCGCTGGAGCACGGTGGGTCCCCAGCGGCAGCATTTGTTGACTTCGTGAATGATGTCAAGATTTCCAAGCTAGATGTTATTTACCCTTT

(16)>Vanillin dehydrogenase (VDH); Ta.2955.2.A1_at; BJ215438

GCCAGAGCGGTTACTACATCGAGCCCACGGTTTTCACCGATGTTAAGAATGACATGATCATAGCAAAGGAGGAGATCTTCGGACCCGTCATGTGCCTGATGAAGTTCAGGACGGTGGAGGAGGCGATCGCGAGGGCCAACGACACGAGGTACGGGCTGGCGGCGGGGGTGGTGACCAAGGACATCGACGTGGCCAACAGGATGACGCGGTCGATCCGCGCCGGGGTGGTGTGGGTCAACTGCTACTTCGCCATGGACAGCGACTGCCCGTTCGGGGGCCGCAAGATGAGCGGTTTCGGCAAGGACGACGGCATGCATGCGCTCGACAAGTTCCTCGCCGTCAAGTCCGTCGTCACCCCCGTCTACGACTCGCCCTGGCTCTAGGCTACATCTCTCTGGCTTGGTTTGCTTCTCTTGTCTGTGCACAGTTAGTTACCAGGAGAAGCAGCAAGGAAGAGAAGGTGAGAAACGTTGTTCGTTGACAGGCAATGT

(17)>Vanillin synthase (VS); TaAffx.107168.1.S1_x_at; CA704943

CCGNGGTCTCTTTCACNCTCANCAGNTGGTTCACTGNGCCCCTTGATNCAATAATTTCGGGTGCANTGGAGGCCTTCCATCCCAGGCATTTGAGTACATCAAGTACAATGNANGGATCGACACCGAGGAGTCCTACCCTTACAAGGGCGTCAATGGCGTCTGCCANTACAAGGGTGAAAATGCCGTATTTCACGTTTTGGACTCAGTTNACACCACACTGAATGCTGNGGACNACCTGAAAAACGCCNNCNGGTTGGTTCGCCCAGTGAGTGTTGCCTTTGAGGTGATCGACGGTCTCAGGNAGTACAAGAGCGNAGTTTACACTANCNCCCATTGTGGNACTACACCTTANGACGTCAACCANGCNCTCTTGGGAGGCCGGGGTACAGTTNTTNGNAACATGCGNGTCCCCNTATTGNNTTCAATCNNANAAACTCNT
